# Generation and molecular characterization of pancreatic cancer patient-derived xenografts reveals their heterologous nature

**DOI:** 10.18632/oncotarget.11530

**Published:** 2016-08-23

**Authors:** Jaeyun Jung, Cue Hyunkyu Lee, Hyang Sook Seol, Yeon Sook Choi, Eunji Kim, Eun Ji Lee, Je-Keun Rhee, Shree Ram Singh, Eun Sung Jun, Buhm Han, Seung Mo Hong, Song Cheol Kim, Suhwan Chang

**Affiliations:** ^1^ Department of Biomedical Sciences, University of Ulsan College of Medicine, Seoul, Korea; ^2^ Department of Physiology, University of Ulsan College of Medicine, Seoul, Korea; ^3^ Department of Convergence Medicine, University of Ulsan College of Medicine, Seoul, Korea; ^4^ Asan Institute for Life Sciences, Asan Medical Center, Seoul, Korea; ^5^ Department of Chemistry, Seoul National University, Seoul, Korea; ^6^ Department of Medical Informatics, College of Medicine, The Catholic University of Korea, Seoul, Korea; ^7^ Mouse Cancer Genetics Program, Center for Cancer Research, National Cancer Institute, Frederick, MD, USA; ^8^ Department of Pathology, Asan Medical Center, Seoul, Korea; ^9^ Department of Surgery, Asan Medical Center, Seoul, Korea

**Keywords:** pancreatic cancer, patient-derived xenograft, single nucleotide polymorphism, cancer panel, heterogeneity

## Abstract

Pancreatic ductal adenocarcinoma (PDAC) is the most challenging type of cancer to treat, with a 5-year survival rate of <10%. Furthermore, because of the large portion of the inoperable cases, it is difficult to obtain specimens to study the biology of the tumors. Therefore, a patient-derived xenograft (PDX) model is an attractive option for preserving and expanding these tumors for translational research. Here we report the generation and characterization of 20 PDX models of PDAC. The success rate of the initial graft was 74% and most tumors were re-transplantable. Histological analysis of the PDXs and primary tumors revealed a conserved expression pattern of p53 and SMAD4; an exome single nucleotide polymorphism (SNP) array and Comprehensive Cancer Panel showed that PDXs retained over 94% of cancer-associated variants. In addition, Polyphen2 and the Sorting Intolerant from Tolerant (SIFT) prediction identified 623 variants among the functional SNPs, highlighting the heterologous nature of pancreatic PDXs; an analysis of 409 tumor suppressor genes and oncogenes in Comprehensive Cancer Panel revealed heterologous cancer gene mutation profiles for each PDX-primary tumor pair. Altogether, we expect these PDX models are a promising platform for screening novel therapeutic agents and diagnostic markers for the detection and eradication of PDAC.

## INTRODUCTION

Pancreatic cancer is a fatal disease in humans [1, 2] and is often referred to as being a silent killer because in general, there are no symptoms until late tumor stages, at which point the tumor cells have metastasized and multiple lesions are formed [3]. Consequently, only 20% of the tumors are resectable [4], which limits translational research using cancer specimens. Currently, a few chemotherapeutic options are available for pancreatic cancer, such as Gemcitabine or fluorouracil (5-FU). However, these are not effective (extending the survival by only a few months) and produce substantial side effects [1, 5]. Therefore, there is an urgent clinical need for the development of novel diagnostic and therapeutic options.

The establishment of a preclinical model for pancreatic cancer is a prerequisite for developing new treatments. A genetically engineered mouse model is currently available for pancreatic cancer, in which activated Kras and/or Trp53 mutant proteins are specifically induced in the pancreatic ductal epithelial cells [6, 7]. However, this model cannot fully reflect human pancreatic cancer, which is genetically heterogeneous. Consequently, the use of patient-derived xenograft (PDX) models is becoming an attractive option because the tumor specimens are directly transplanted into immuno-compromised mice, providing a faithful representation of individual tumors [8]. However, establishing PDX can also be a challenge, with the success rate varying according to several factors, including the type of tumor, recipient mouse, transplant technique, and time gap between surgery and transplantation [9].

Recent studies have successfully generated PDXs for pancreatic cancer. For example, Helene et al. described 12 PDAC PDXs and PDX-derived cell lines that showed sonic hedgehog (SHH) signaling activation [10]. Delitto et al. also successfully generated 15 PDXs from 25 specimens and demonstrated that they had a conserved histology with the primary tumors [11]. Furthermore, they also found that mouse stromal cells infiltrated the human cancer cells, suggesting active tumor-stromal interactions in pancreatic cancer. Regarding the molecular analysis of PDAC PDXs, Matti et. al. reported KRAS and PIK3CA mutation analysis up to eight passages and found a similar mutation frequency in PDXs [9]. Therefore, it seems that use of the PDX model is very successful for pancreatic cancer, suggesting that it would be a good preclinical model for understanding this complex disease.

Here, we describe 20 pancreatic PDXs originating from PDAC patients who underwent surgery at the Asan Medical Center, Seoul, Korea. Clinical information and analysis of the molecular data revealed that these pancreatic PDXs have novel and heterologous characteristics.

## RESULTS

### Generation of pancreatic patient-derived xenografts (PDXs) and primary cells

**Figure 1:**
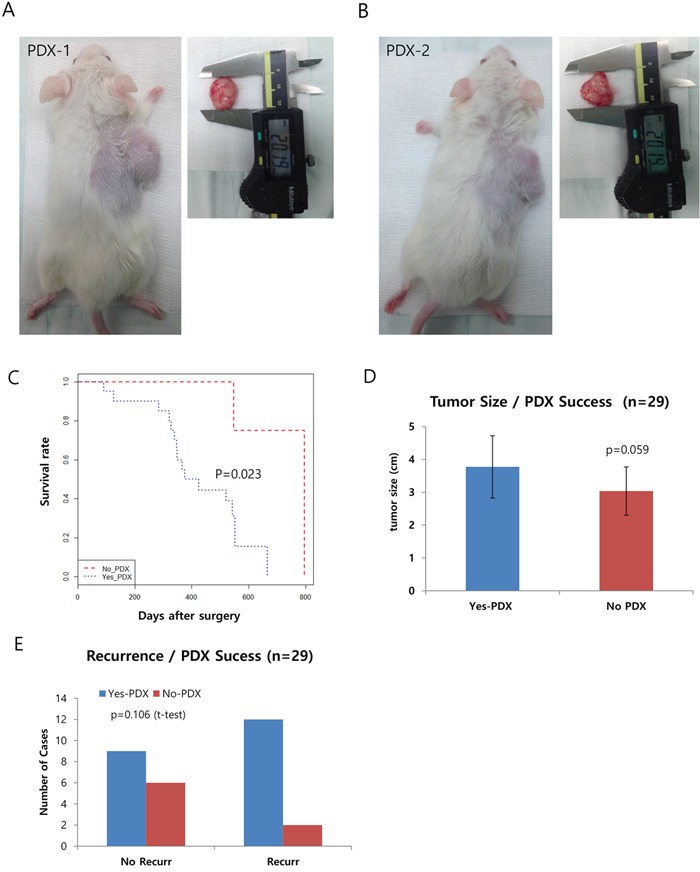
Generation of pancreatic patient-derived xenografts (PDXs) and the clinical features affecting their success **A-B.** Representative pictures of PDXs in NOD/SCID mice. The right panels show the dissected tumors being measured with calipers. **C.** Kaplan-Meyer curve of the two groups of successful xenograft (Yes PDX in Blue) or failed xenograft (No PDX in Red). **D** and **E.** Graphs showing a positive correlation between the success of the xenograft and other clinical factors, including survival (D) and recurrence (E).

In total, we obtained 29 freshly dissected specimens from surgery, in which we carefully selected the region that exhibited enriched tumor cells. Approximately1 cm^3^ of tumor tissue was obtained and cut into small pieces (1–2mm^3^on average). Three or four of these pieces were then subcutaneously transferred into NOD/SCID mice under anaesthetized conditions. From here, it usually takes 1~2 months for the tumor to grow. Using this method, we successfully produced 20 PDXs, representing a 72.4% success rate. Representative pictures of these PDXs are shown in Figure 1A and 1B, and Supplementary Figure S1. We also obtained six primary cancer cell lines from the PDXs (Supplementary Figure S2; also see Supplementary Table S1 for clinical information) and utilized some of these cells in our genomic analysis, along with human pancreatic ductal epithelial (HPDE) cells and PDAC (Panc1) cells.

### Analysis of clinical data reveals several criteria affecting the success of PDX

Next, we checked the clinical information to determine which factor(s) affected the success of PDX (see Table 1 for a summary). Due to the highly metastatic properties of PDAC, most of our PDX samples fell into Stage IIA or IIB, exhibiting lymph node metastasis but not distance metastasis. Here, we specifically focused on tumor size at surgery, recurrence, gender, and survival/death of the patient. Other factors such as lymphatic/vascular invasion, histological type, and distant metastasis were not considered due to the limited number of cases for each. Among the clinical characteristics analyzed, we found survival/death of the patient was significantly associated with the success rate of PDX (*P*=0.023 by Cox proportional hazard regression analyses, Figure 1C). In addition, tumor size (*P*=0.059) and recurrence showed a positive correlation but was not significant (Figure 1D and 1E). Multivariate analysis of the recurrence and tumor size, however, revealed the tumor size is a significant factor for the success of PDX (p=0.048, Table 2).

**Table 1:** Clinical characteristics of the parental tumors of the patient-derived xenografts (PDXs)

ID	Tumor size after surgery	pT	pN	M	M1 Site	Stage	Lymphatic Invasioninvasion	Vascular Invasioninvasion	Histologic_type	Recur	Recur_type	Death_data	Age	Gender
AMC001	3.5	3	0	0		IIA	1	1	DUCTAL ADENOCARCINOMA	1	Remnant pancreas	2014-04-08	66	M
AMC002	2.1	3	0	0		IIA	0	0	DUCTAL ADENOCARCINOMA	1	Liver meta	2014-09-26	30	M
AMC003	3.5	3	1	0		IIB	0	0	DUCTAL ADENOCARCINOMA				66	M
AMC004	3.1	3	1	0		IIB	0	0	DUCTAL ADENOCARCINOMA				53	M
AMC005	5.7′	3	1	0		IIB	1	1	DUCTAL ADENOCARCINOMA	1	Liver meta	2014-12-01	70	M
AMC006	2.2	3	0	0		IIA	0	0	DUCTAL ADENOCARCINOMA	1	Encasing the celiac trunk and SMA	2015-05-30	58	F
AMC007	4.3	3	1	0		IIB	1	1	ADENOCARCINOMA	1	Remnant pancreas(tail)		53	M
AMC008	3.5	3	0	0		IIA	1	1	ADENOCARCINOMA, AOV				50	M
AMC009	3.7	3	1	0		IIB	1	1	DUCTAL ADENOCARCINOMA	1	Liver meta	2015-02-20	57	M
AMC010	4.3	3	1	0		IIB	1	1	DUCTAL ADENOCARCINOMA	1	Liver meta	2015-04-26	48	F
AMC011	3.7	3	1	0		IIB	1	1	DUCTAL ADENOCARCINOMA	1	Liver meta		61	M
AMC012	5.5	3	0	0		IIA	0	0	DUCTAL ADENOCARCINOMA	1	Remnant pancreas(head)	2015-03-21	64	F
AMC013	5.1	3	1	0		IIB	1	1	DUCTAL ADENOCARCINOMA			2015-03-15	50	M
AMC014	5	3	1	0		IIB	1	1	SARCOMATOID CARCINOMA	1	Liver meta c peritoneal seeding	2014-07-22	59	F
AMC015	3.6	3	1	0		IIB	1	1	DUCTAL ADENOCARCINOMA			2015-04-06	73	F
AMC016	3.5	3	1	1	liver	IV	1	1	DUCTAL ADENOCARCINOMA				61	M
AMC017	4.5	3	1	0		IIB	0	0	DUCTAL ADENOCARCINOMA			2015-04-29	56	M
AMC018	3.7	3	0	0		IIA	0	0	DUCTAL ADENOCARCINOMA				50	M
AMC019	2.1	3	1	0		IIA	1	1	DUCTAL ADENOCARCINOMA	1	Hepatoduodenal ligament and around pancreaticojejunostomy site	2015-03-26	71	F
AMC020	3.9	3	0	0		IIA	0	0	ADENOSQUAMOUS CARCINOMA	1	Liver meta	Lost	77	F

**Table 2:** Summary of the multivariate analysis affecting the success rate of PDX

	Coefficient	Std. error	p-value
**Tumor_size**	1.8052	0.9135	0.0481
**Recur**	2.6846	1.4763	0.069
**(intercept)**	−6.0514	3.2551	0.063

For the tumor size and recurrence, a logistic regression method was used to determine the effect of multiple clinical factors on the success of PDX.

### Comparison of the histological features of the PDX and its original primary tumor

**Figure 2:**
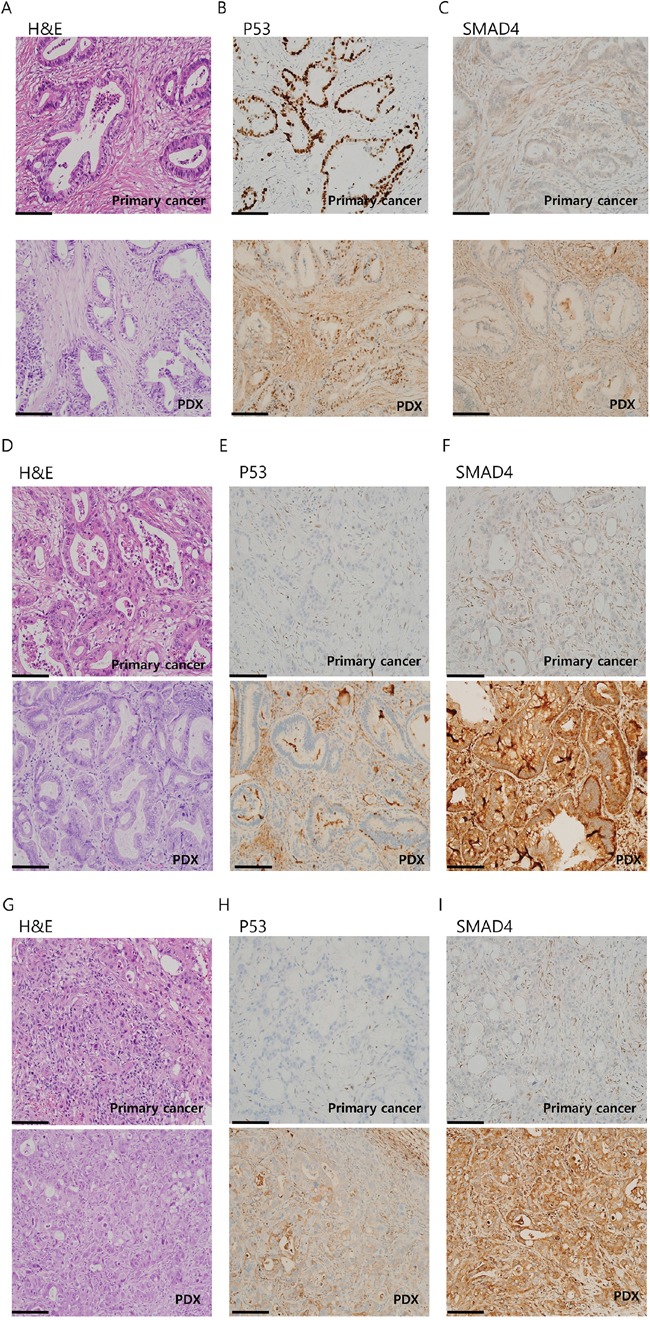
Immunohistochemistry (IHC) analysis of patient-derived xenograft (PDX)-primary tumor pairs reveals a conserved histology IHC images of three PDX-primary tumor pairs: AMC001 **A-C.** AMC002 **B-D.** and AMC003 **E-G.** stained with hematoxylin and eosin (H&E) (A, D, G), P53 (B, E, H), or SMAD4 (C, F, I). Scale bar:100μm.

To confirm that the gross histology of the primary tumor was conserved in the PDXs, we performed hematoxylin and eosin (H&E) staining and immunostaining with anti-P53 and SMAD4 antibodies. These data are summarized in Table 3. Overall, we observed similarities between the gross histology of the primary tumors and PDX tumors (Figure 2A, 2D, and 2G, and Supplementary Figure S3). In addition, P53 and SMAD4 staining showed a comparable reactivity in most cases (13 out of 16) of PDX-primary tumor pairs (Figure 2B, 2C, 2E, 2F, 2H, and 2I, and Supplementary Figure S3). These results show that the PDAC PDXs generated in this study recapitulated the primary tumors histologically.

**Table 3:** List of genes containing deleterious variants that are frequently found in pancreatic patient-derived xenografts (PDXs)

All									
[1]	[2]	[3]	[4]	[5]	[6]	[7]	[8]	[9]	[10]
*LAMB3*	*TPO*	*MYBPC1*	*CD101*	*SPINK5*	*MOV10L1*	*C3orf20*	*RAET1E*	*TTN*	*PKD1L2*
222	132	132	126	114	110	104	102	98	92
PDX 1									
[1]	[2]	[3]	[4]	[5]	[6]	[7]	[8]	[9]	[10]
*TPO*	*ADAM15*	*MOV10L1*	*TMEM176B*	*PASK*	*DLG1*	*SLC7A9*	*RAET1E*	*PRR5*	*PAK6*
12	12	10	8	8	8	6	6	6	6
PDX 2									
[1]	[2]	[3]	[4]	[5]	[6]	[7]	[8]	[9]	[10]
*TTN*	*CPT1B*	*MYBPC1*	*LAMB3*	*ZNF484*	*TMEM176B*	*FAM129C*	*DLG1*	*ANKRD6*	*RAET1E*
14	14	12	12	8	8	8	8	8	6
PDX 3									
[1]	[2]	[3]	[4]	[5]	[6]	[7]	[8]	[9]	[10]
*TTN*	*LAMB3*	*MOV10L1*	*ZNF484*	*TMEM176B*	*SPINK5*	*SLC7A9*	*RAET1E*	*PCDHGA1*	*NFATC3*
14	12	10	8	8	6	6	6	6	6
PDX 4									
[1]	[2]	[3]	[4]	[5]	[6]	[7]	[8]	[9]	[10]
*TTN*	*CPT1B*	*LAMB3*	*TMEM176B*	*PRICKLE1*	*PASK*	*CX3CR1*	*SPINK5*	*RAET1E*	*PPP2R4*
14	14	12	8	8	8	8	6	6	6
PDX 5									
[1]	[2]	[3]	[4]	[5]	[6]	[7]	[8]	[9]	[10]
*TTN*	*TPO*	*MYBPC1*	*NUP62*	*MOV10L1*	*ZNF484*	*TMEM176*B	*TLR10*	*FAM129C*	*DLG1*
14	12	12	10	10	8	8	8	8	8
PDX 6									
[1]	[2]	[3]	[4]	[5]	[6]	[7]	[8]	[9]	[10]
*CAMKK2*	*NUP62*	*MOV10L1*	*ZNF484*	*PASK*	*FAM129C*	*CX3CR1*	*SPINK5*	*SP110*	*SLC7A9*
18	10	10	8	8	8	8	6	6	6
PDX 7									
[1]	[2]	[3]	[4]	[5]	[6]	[7]	[8]	[9]	[10]
*TMEM176B*	*TLR10*	*SWT1*	*FAM129C*	*SPINK5*	*SLC7A9*	*PAK6*	*LAMB3*	*GFAP*	*CD101*
8	8	8	8	6	6	6	6	6	6
PDX 8									
[1]	[2]	[3]	[4]	[5]	[6]	[7]	[8]	[9]	[10]
*TPO*	*MYBPC1*	*LAMB3*	*DLG1*	*ANKRD6*	*SPINK5*	*SP110*	*RAET1E*	*PMEL*	*NRAP*
12	12	12	8	8	6	6	6	6	6
PDX 9									
[1]	[2]	[3]	[4]	[5]	[6]	[7]	[8]	[9]	[10]
*TPO*	*MYBPC1*	*LAMB3*	*NUP62*	*TLR10*	*PASK*	*FAM129C*	*SPINK5*	*SLC7A9*	*RAET1E*
12	12	12	10	8	8	8	6	6	6
PDX 10									
[1]	[2]	[3]	[4]	[5]	[6]	[7]	[8]	[9]	[10]
*LAMB3*	*TMEM176B*	*TLR10*	*SLC7A9*	*PPP2R4*	*NBR1*	*MUSK*	*EGFLAM*	*CD101*	*CAPG*
12	8	8	6	6	6	6	6	6	6
PDX 11									
[1]	[2]	[3]	[4]	[5]	[6]	[7]	[8]	[9]	[10]
*CAMKK2*	*CPT1B*	*NUP62*	*MOV10L1*	*SWT1*	*PASK*	*FAM129C*	*SLC7A9*	*RAET1E*	*PRR5*
18	14	10	10	8	8	8	6	6	6
PDX 13									
[1]	[2]	[3]	[4]	[5]	[6]	[7]	[8]	[9]	[10]
*TPO*	*LAMB3*	*ADAM15*	*ZNF484*	*TLR10*	*CX3CR1*	*SP110*	*SLC7A9*	*RAET1E*	*NRAP*
12	12	12	8	8	8	6	6	6	6
PDX 14									
[1]	[2]	[3]	[4]	[5]	[6]	[7]	[8]	[9]	[10]
*ZNF484*	*SPINK5*	*SLC7A9*	*PPP2R4*	*NBR1*	*MUSK*	*LAMB3*	*DFNA5*	*CD101*	*C3orf20*
8	6	6	6	6	6	6	6	6	6
PDX 15									
[1]	[2]	[3]	[4]	[5]	[6]	[7]	[8]	[9]	[10]
*TTN*	*CPT1B*	*LAMB3*	*ADAM15*	*NUP62*	*MOV10L1*	*PRICKLE1*	*SPINK5*	*NBR1*	*GBP3*
14	14	12	12	10	10	8	6	6	6
PDX 17									
[1]	[2]	[3]	[4]	[5]	[6]	[7]	[8]	[9]	[10]
*CAMKK2*	*ZNF484*	*TMEM176B*	*SPINK5*	*PPP2R4*	*NBR1*	*MUSK*	*GFAP*	*EGFLAM*	*CHIA*
18	8	8	6	6	6	6	6	6	6
PDX 18									
[1]	[2]	[3]	[4]	[5]	[6]	[7]	[8]	[9]	[10]
*CAMKK2*	*MYBPC1*	*MOV10L1*	*ZNF484*	*TLR10*	*SWT1*	*PRICKLE1*	*FAM129C*	*CX3CR1*	*SPINK5*
18	12	10	8	8	8	8	8	8	6
PDX 19									
[1]	[2]	[3]	[4]	[5]	[6]	[7]	[8]	[9]	[10]
*CAMKK2*	*CPT1B*	*LAMB3*	*MOV10L1*	*ZNF484*	*TLR10*	*SPINK5*	*RAET1E*	*NRAP*	*MUSK*
18	14	12	10	8	8	6	6	6	6
PDX 20									
[1]	[2]	[3]	[4]	[5]	[6]	[7]	[8]	[9]	[10]
*TTN*	*MYBPC1*	*LAMB3*	*ZNF484*	*TMEM176B*	*DLG1*	*SP110*	*PRR5*	*PAK6*	*NBR1*
14	12	12	8	8	8	6	6	6	6
HPDE									
[1]	[2]	[3]	[4]	[5]	[6]	[7]	[8]	[9]	[10]
*ZZZ3*	*ZZEF1*	*ZYX*	*ZYG11A*	*ZXDC*	*ZSWIM6*	*ZSWIM4*	*ZSWIM2*	*ZSCAN5B*	*ZSCAN5A*
0	0	0	0	0	0	0	0	0	0
Pancl									
[1]	[2]	[3]	[4]	[5]	[6]	[7]	[8]	[9]	[10]
*MOV10L1*	*TLR10*	*SWT1*	*CX3CR1*	*SPINK5*	*RAET1E*	*NBR1*	*GBP3*	*CHIA*	*ZNF229*
10	8	8	8	6	6	6	6	6	4

### An exome single nucleotide polymorphism (SNP) array enables grouping of the PDXs and identifies putatively functional SNPs

To characterize the PDXs at the molecular level, we performed an exome SNP array. Among the 20 PDXs, we excluded #12 and 16 due to the poor data quality. Instead, we included primary cancer cell line (59390), HPDE cells and Panc1 cells as cancer and normal cell controls. We aimed to compare the SNP profile of each sample so that we could subcategorize PDXs, and discover putatively functional SNPs. These functional SNPs could help us to better understand the molecular mechanisms of tumorigenesis as well as tumor heterogeneity.

**Figure 3:**
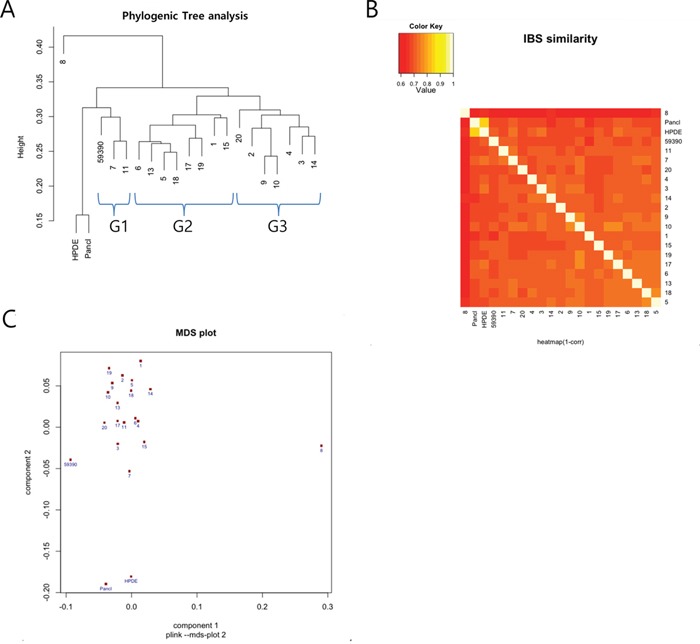
Summary of a single nucleotide polymorphism (SNP) array analysis from 18 patient-derived xenografts (PDXs), a primary tumor cell line (59390), pancreatic ductal adenocarcinoma (Panc1) cells, and human pancreatic ductal epithelial (HPDE) cells The results were obtained from 623 deleterious cancer-specific SNPs. **A.** Phylogenetic tree showing three main clusters of the variants (marked G1, G2, and G3) occurring among the PDXs. **B.** Information-Based Similarity (IBS) matrix based on the SNP variants among the PDXs. **C.** Multidimensional scaling (MDS) plot showing a clustering pattern.

We first selected 24,000 non-rare variants from 244,770 variants using plink (option – maf 0.1). We then found 1,385 deleterious variants, as predicted by Polyphen2 and Sorting Intolerant from Tolerant (SIFT) (see methods). Following this, we removed variants whose risk alleles were present in HPDE to obtain only cancer-specific variants, which left us with 623 variants (Supplementary Table S2). Table 3 summarizes the top 10 genes for each PDX that showed a high number of deleterious variants. We found that there was little overlap between these variants among the PDXs, with the sum of the top 10 variants for each PDX tumor comprising only a minor portion, ranging from 62 (10.9%) to 102 (16.4%), which implied that pancreatic PDXs are heterogeneous. However, a phylogenetic tree analysis of the functional SNPs yielded three groups of clusters (Figure 3A). An Information-Based Similarity (IBS) matrix analysis of the deleterious SNPs (Figure 3B) and a multidimensional scaling (MDS) plot analysis (Figure 3C) showed 70~80% similarity (with the exception of #8), confirming the diversity of genetic variants among the pancreatic PDXs.

### Comprehensive Cancer Panel reveals unknown genetic alterations specific to pancreatic cancer

Although the data shown in Figure 3 and Table 3 generated by the exome SNP array provided useful information to classify the18 PDXs along with the primary cancer cell lines, they were insufficient for determining the molecular characteristics of the PDX-primary tumor pairs in terms of cancer-related genes. Therefore, to examine how the cancer-related mutations were conserved between the PDXs and primary tumors, we conducted an analysis of eight PDX-primary tumor pairs using Ion Ampliseq Comprehensive Cancer Panel, which covers 409 cancer-related genes (Supplementary Figure S4 for general data; for the gene list, see Thermofisher.com). The total number of variants was 40,827, of which 10,031 were novel (Supplementary Table S3). There were up to 1,804 variants in the coding region and untranslated region (UTR), and 13 of the genes with these variants were predicted to be highly affected by them. Table 4 shows examples of the variants that had a large impact. Notably, we found that PTEN, SMAD4, and TP53 were in this list, confirming previous findings [12, 13] (for raw data, see Supplementary Table S4).

**Table 4:** Examples of variants with a high impact, as identified from the Comprehensive Cancer Panel analysis

CHROM	POS	REF	ALT	Variant type	QUAL	DP	Allele	Effect	Impact	Gene_Name	HGVS.c	HGVS.p
chr5	55243415	G	A	SNP	535.96	1513	A	stop_gained&splice_region_variant	HIGH	`IL6ST	c.1843C>T	p.Gln615*
chr3	37818889	C	T	SNP	43280.76	29332	T	stop_gained	HIGH	`ITGA9	c.2548C>T	p.Gln850*
chr9	134073362	A	AC	INS	4361.47	934	AC	frameshift_variant	HIGH	`NUP214	c.4484_4485insC	p.Glu1495fs
chr10	89720633	CT	C, CTT	DEL, INS	25093.71	7744	T	splice_acceptor_variant&intron_variant	HIGH	`PTEN	c.802-3dupT	
chr8	145738599	A	C	SNP	241.71	1903	C	splice_donor_variant&intron_variant	HIGH	`RECQL4	c.2463+2T>G	
chr18	48573537	G	T	SNP	22955.8	30701	T	stop_gained	HIGH	`SMAD4	c.121G>T	p.Glu41*
chr17	7579470	C	CG	INS	6935.51	6514	CG	frameshift_variant	HIGH	`TP53	c.216dupC	p.Val73fs

SNP: Single nucleotide polymorphism; INS: insertion; DEL: deletion; HGVS: Human Genome Variation Society

**Figure 4:**
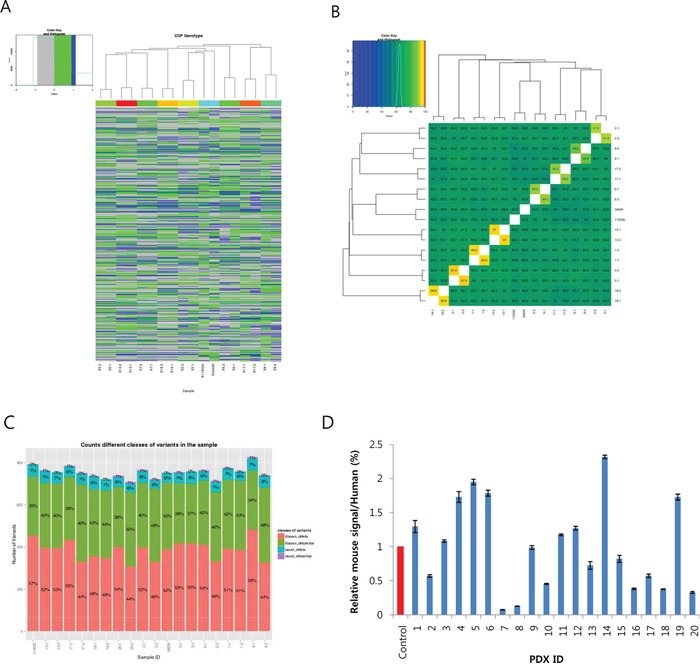
Summary of the Ion-Ampliseq Comprehensive Cancer Panel analysis for eight patient-derived xenograft (PDX)-primary tumor pairs **A.** Cluster analysis of 18 samples (8 pairs and two cell lines) based on the variants found in 402 cancer genes. **B.** Similarity matrix showing conservation of the variants between PDX and primary tumors ranging from 90.5% to 97.4%. **C.** Number of variants found in each PDX and primary tumor sample. The numbers in the bar denote the proportion of known/novel and homologous/heterologous variants, respectively. **D.** Estimation of the proportion of infiltrated mouse cells in the PDXs calculated by dividing mouse RP13a expression by humanRP13a expression. The control was 5% mouse cells mixed with human pancreatic ductal epithelial (HPDE) cells.

Clustering analysis (Figure 4A) showed that there was a high similarity between each PDX and primary tumor, with the exception of PDX #20. Furthermore, in the similarity matrix (Figure 4B), we could clearly see the conservation of most cancer gene variants between each pair of PDX-primary tumors (ranging from 90.2% to 97.4%). Interestingly, however, all other combinations among the 18 primary tumors showed much less similarity (from 59% to 67.7%), suggesting heterogeneity of the PDX tumors. The numbers of variants found in the tumors were very close to each other (around 700; Figure 4C and Supplementary Table S5, column F), implying that there was comparable genetic alteration among the tumors. This was further confirmed by counting the number of novel variants (Supplementary Table S6).

Lastly, we measured the degree of mouse cell infiltration by measuring the relative mouse RPL13a expression to human RPL13a in the PDXs. We also included a control comprised of 95% HPDE mixed with 5% mouse fibroblast cells. This showed that there was 1–12% mouse RPL13a expression (Figure 4D), suggesting variable mouse cell infiltration in the PDXs.

### Western blot analysis of PDXs for the major growth signaling/cell cycle regulatory proteins reveals their heterologous nature

**Figure 5:**
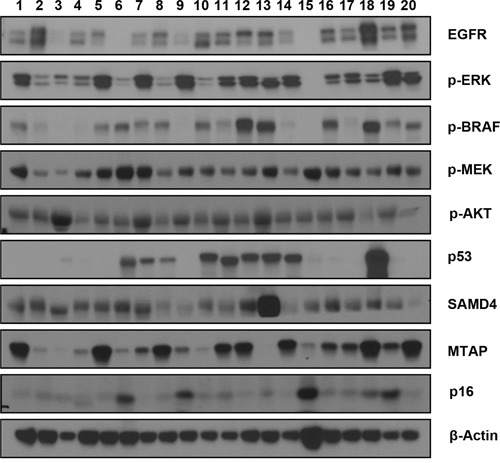
Western blots showing the expression levels of various growth signaling and cell cycle regulatory proteins In total, 20 pancreatic patient-derived xenografts (PDXs) were analyzed. The name of each protein is marked on the right. The beta-actin antibody was used to ensure equal loading.

In addition to the genetic analyses described above, which used an SNP array and Comprehensive Cancer Panel, we performed a series of western blot analyses to check the levels of the major growth signaling and cell cycle regulatory proteins that have previously been implicated in pancreatic cancer [14–17]. Accordingly, we found heterogeneous expression levels of these proteins (Figure 5). In particular, we observed the frequent loss of TP53 expression (by approximately 50%), as well as the minimal expression of P16. In contrast, we detected various levels of p-BRAF and p-MEK, which are major downstream effectors of K-Ras [18]. Interestingly, some of the PDXs (#4, 9, and 15) showed discordant p-BRAF and p-MEK levels, suggesting that some alternative pathway activates p-MEK in these tumors. We detected a relatively consistent level of p-AKT and SMAD4, whereas the levels of p-ERK and MTAP varied greatly. Therefore, our protein analysis revealed that the PDXs have a heterologous molecular nature that resembles the known heterologous character of primary tumors [19], supporting the strategy of using PDX as a preclinical model in pancreatic cancer.

## DISCUSSION

Xenograft transplantation of human PDAC cells or tissues was first performed in the late 1990s [20], with subsequent studies reporting a high degree of similarity between the PDXs and primary cancer cells, and passage-dependent genetic changes [9]. A recent study using 96 PDAC patient samples estimated the frequency of mutations in a panel of 22 cancer predisposition genes, which led to the identification of 14 pathogenic mutations in 13 patients (13.5%) [21]. Other studies on pancreatic cancer xenografts have analyzed gene expression and/or copy number variations, but have discovered only small numbers of genetic variants [9, 22, 23]. Therefore, our report provides more information about the potentially deleterious variants to pancreatic cancer research field.

In our SNP analysis, we initially found 762 deleterious (as predicted by SIFT and Polyphen2) variants in HPDE. Since we included HPDE as a non-cancer cell control, we subtracted these variants from the total variants to obtain the number of cancer-specific deleterious variants. However, we cannot exclude the possibility that this subtraction might have missed some variants that is functional in cancer cells. The list of top-ranked genes (which shows frequent SNPs in multiple PDXs) included a number of promising candidates for functional analysis. For example, *LAMB3*, the top-ranked gene, produces lamininb3, which is one of the major components of the extracellular matrix (ECM) of pancreatic cancer [24], and these variants generate diverse types of missense mutations, whose function needs to be further analyzed. By contrast, *CD101*, which has been reported as a potential risk-associated variant for PDAC [25], plays a role as an inhibitor of CD3-induced T-cell proliferation [26], and so the variants of this gene may have the immuno-modulatory effect on cancer cells. Further molecular study will reveal the exact function of these variants in pancreatic cancer.

Because different number of samples were analyzed in SNP array and CCP (18 PDXs in SNP, 8 primary tumor-PDX pairs in CCP), a direct comparison of the clustering result from the two analysis was not possible. However, we were able to compare several samples analyzed in both platforms. For example, we could see the #5/#18 and #6/13 pairs are closely related in the Group 2 of SNP data (Figure 3A) but only #5/#18 pair is closely related in the CCP analysis (Figure 4A). Therefore, we think the results of the two techniques are only partly matching. The possible reason of this result might be the difference of analytical platform. Specifically, the SNP array covers about 20,000 exome SNPs throughout the genome but the CCP covers only around 400 cancer genes.

Taken together, our findings indicate that the PDX model can provide a faithful representation of patient tumors. Furthermore, these PDXs retained the heterologous nature of pancreatic cancer cells, enabling us to use this model for preclinical research, as well as the basic study of this disease.

## MATERIALS AND METHODS

### Tumor implantation into mice

The animal care protocol for this study was approved by the International Animal Care and Use Committee (IACUC) of the Laboratory of Animal Research at the Asan Medical Center, Seoul, Korea. Five-week-old male NOD/SCID mice were used for tumor engraftment and were grown in a specific pathogen-free facility. The surgical specimens were obtained under permission from the institutional review board (IRB) of the Asan Medical Center (No. S2013-0744-0009).

Fresh tumor tissues were obtained from pancreatic cancer patients who underwent surgery and were immediately placed in RPMI medium (10% FBS, 1% penicillin/streptomycin) at 4°Cin the refrigerator. As soon as possible after this, the samples were spliced into one to two 2-mm^3^ fragments and implanted into the interscapular fat pad of the mice subcutaneously. All of the animals were anesthetized with 15 mg/kg of Zoletil® (Virbac, USA) and 2.5 mg/kg of Rompun® (Bayer Korea, Korea) by intraperitoneal injection for tumor implantation. Following implantation, the mice were monitored twice per week for at least 12 months. Once the xenograft tumor had attained a size of 300–500 mm^2^, the tumor was excised and the mice were euthanized following the protocol of the Laboratory of Animal Research at the Asan Medical Center. Part of the tumor that had been excised from the mouse was then engrafted into another NOD/SCID mouse for expansion, while the residual part of the tumor was placed in a freezing medium with dimethyl sulfoxide (DMSO) and kept in a deep freezer.

### Immunohistochemical staining

Tumors were fixed in 10% formalin for at least 24hand then embedded in paraffin. Both human and mouse tumor tissues were sectioned at a 5μm thickness and stained with H&E. Immunohistochemistry(IHC) was performed to examine the expression of p53 and DPC4 in the primary human tumors, as previously described [27], following the protocol of the Department of Diagnostic Pathology at the Asan Medical Center. Briefly, after deparaffinization and antigenic retrieval, the slides were labeled with a monoclonal antibody against p53 (cloneDO-7, 1:3,000; DAKO, Glostrup, Denmark) and DPC4 (clone EP618Y, 1:100; GeneTex, Irvine, CA, USA). Labeling was detected using the avidin-biotin complex staining method. 3, 3′-diaminobenzidine (DAB) was used as the chromogen for p53and 3-amino-9-ethylcarbazole was used for DPC4. A pathologist who was experienced in pancreatic cancer reviewed the slides to compare the tumor architecture and desmoplastic appearance.

### Collection of exonic variants

Genetic variant data for the PDX samples were gathered using the InfiniumHumanExomee12 v1.2 BeadChip. This platform targets putative functional exonic variants selected from over 12,000 individual exome and whole-genome sequences. The output data contain both SNP and single base insertion or deletion information. The data also include the GeneCall score for each variant of the samples, which is a quality control measure that was scaled between 0 and 1.

### Quality control for genetic data

For each sample, we counted the number of variants that completely failed in genotype calling (GeneCall score = 0) (Supplementary Table S7). This resulted in the exclusion of two samples(#12 and 16) that had an exceedingly large number of failed genotypes (>10,000). We then chose 217,793 variants (from a total of 244,770) that had a positive GeneCall score in all remaining 21 samples, and used these variants in the subsequent genetic analysis.

### Genetic similarity and MDS analysis

We used plink v1.07to perform a similarity analysis using the genetic data. We calculated the identify-by-state (IBS) pairwise similarity between samples using the--cluster-distance-matrix options in plink. We then generated a heatmap and dendrogram using R. We also generated an MDS plot using the--cluster--mds-plot options in plink and the R package heatmap v3.

### Prediction and selection of deleterious variants

We used SIFT [28] and Polyphen2 [29] to predict and select putatively important variants that may cause protein damage. Polyphen2 predicted which variants were possibly damaging, probably damaging, or benign, while SIFT predicted which variants were damaging or tolerated based on the Rapid Stain Identification Series (RSID) of each variant. We defined a variant as deleterious if the Polyphen2 prediction was possibly/probably damaging or if the SIFT prediction was damaging. Among 244,770 variants (i.e., all variants before applying the quality controls), 13,613 were predicted as being deleterious.

### Defining the gene disruption variable

To analyze the data at the gene level, we newly defined a genetic variable that indicated whether the gene was disrupted or not. We defined a gene as being disrupted if any variant that was predicted as being deleterious within the gene carried the risk allele. Since Polyphen2 and SIFT did not provide information about the risk allele, we obtained this information from Illumina, and confirmed this by comparing the data to the predictions from Ensemble.

### Western blot analysis

Western blot analysis was performed as previously described [30]. Briefly, cells were lysed in lysis buffer (150 mM NaCl, 1% Triton X-100, 1% sodium deoxycholate, 50 mMTris-HCl [pH 7.5], 2 mM ethylenediaminetetraacetic acid [EDTA;pH 8.0], and 0.1% SDS). Following this, 10~50 μg of protein were separated on SDS PAGE, transferred to a nitrocellulose membrane, and probed with anti-EGFR, p-ERK, p-BRAF, p-MEK, p-AKT, P53, SMAD4, MTAP, and p16 (1:1,000; Cell Signaling Technology). The membranes were then stripped and re-probed with anti β-actin antibody (1:1,000; Santa Cruz Biotechnology, CA, USA) to ensure equal loading.

### Statistics

For the analysis of clinical factors affecting successful xenograft, we applied a univariate and multivariate statistical models. For univariate statistical analysis, the statistical significance was measured by a t-test or a chi-square test. For multivariate analysis, a logistic regression method was used to determine the effect of multiple clinical factors. The survival curve was plotted using Kaplan-Meier method and the significance of the differences between the two curves was calculated by a log-rank test. Cox proportional hazards regression model was also used both for individual variable and for the multivariate analysis. All the statistical analysis was also carried out by Microsoft Excel or the R package (ver.3.3).

## SUPPLEMENTARY FIGURES AND TABLES




