# Supplementary material for: Generation and molecular characterization of pancreatic cancer patient-derived xenografts reveals their heterologous nature
Source: Oncotarget. 2016 Aug 23;7(38):62533–46. doi: 10.18632/oncotarget.11530 (PMC5308744; doi:10.18632/oncotarget.11530)
Supplement: Supplementary file 1 [file oncotarget-07-62533-s001.pdf]

Generation and molecular characterization of pancreatic cancer patient-derived xenografts reveals their heterologous nature

SUPPLEMENTARY FIGURES AND TABLES

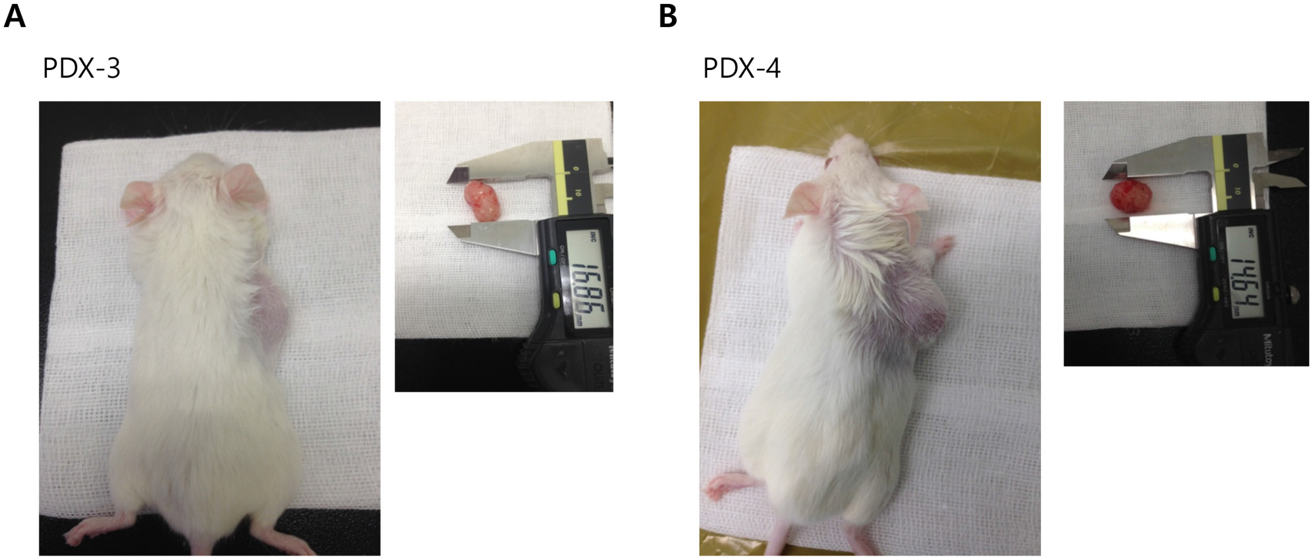

Supplementary Figure S1: Examples of pancreatic patient-derived xenografts (PDXs) generated in NOD-SCID mice. Left: Before dissection; right: tumor in calipers after dissection.

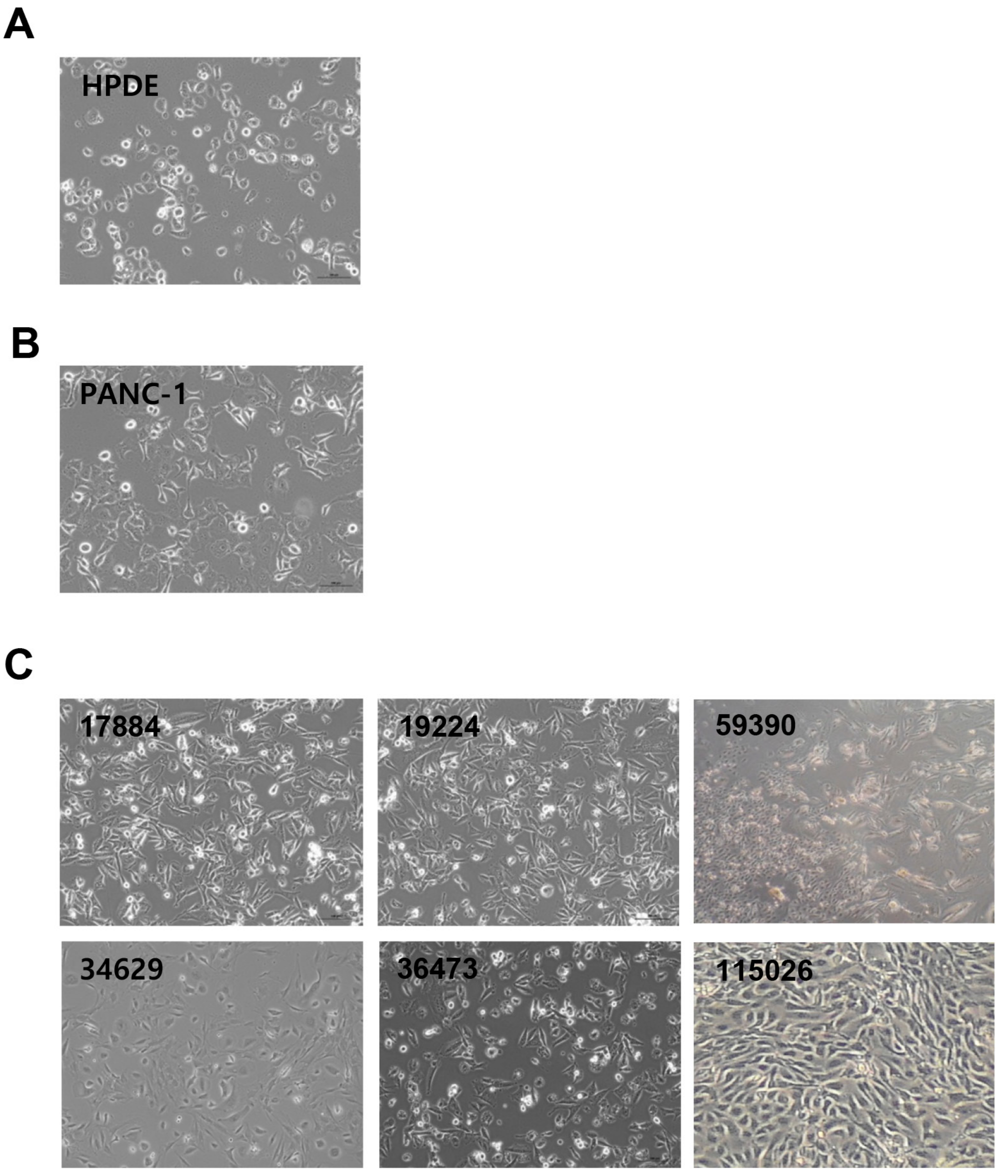

Supplementary Figure S2: Representative picture of primary cancer cells derived from patient-derived xenografts (PDXs) and cell lines. **A.** Human pancreatic ductal epithelial cell (as a normal control). **B.** Panc1 pancreatic ductal adenocarcinoma cell (as a cancer control). **C.** Human pancreatic primary cancer cells from PDX tissues.

A

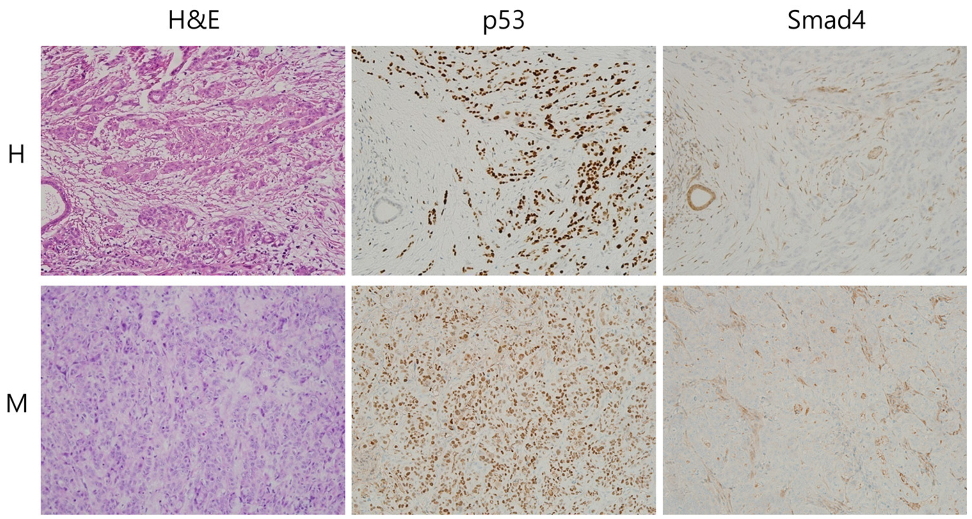

B

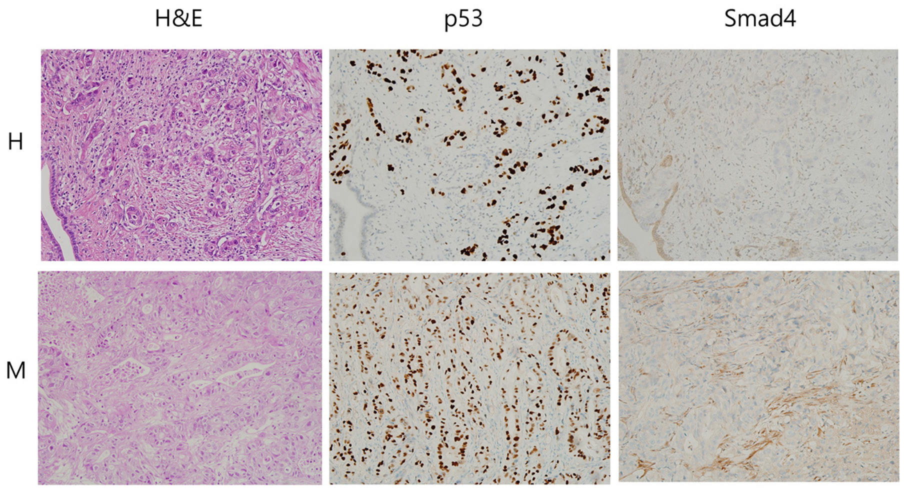

C

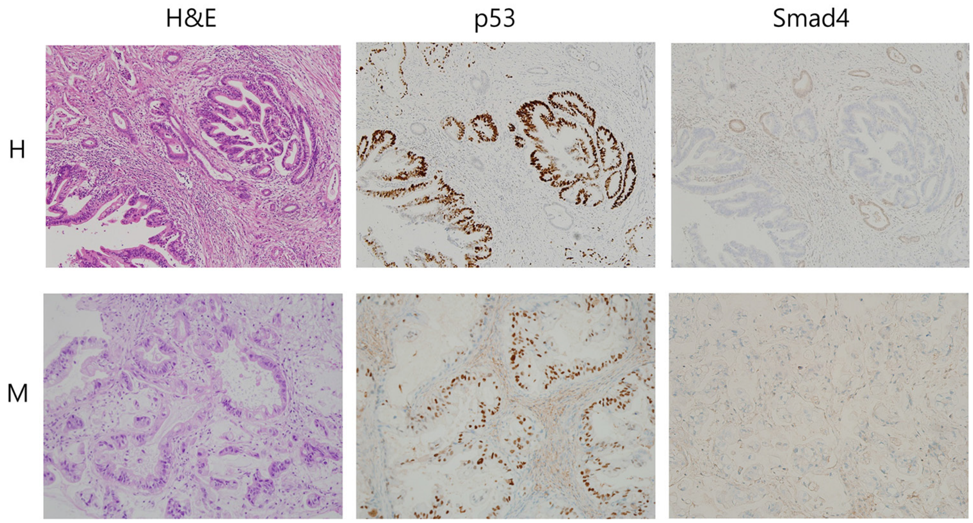

(Continued)

D

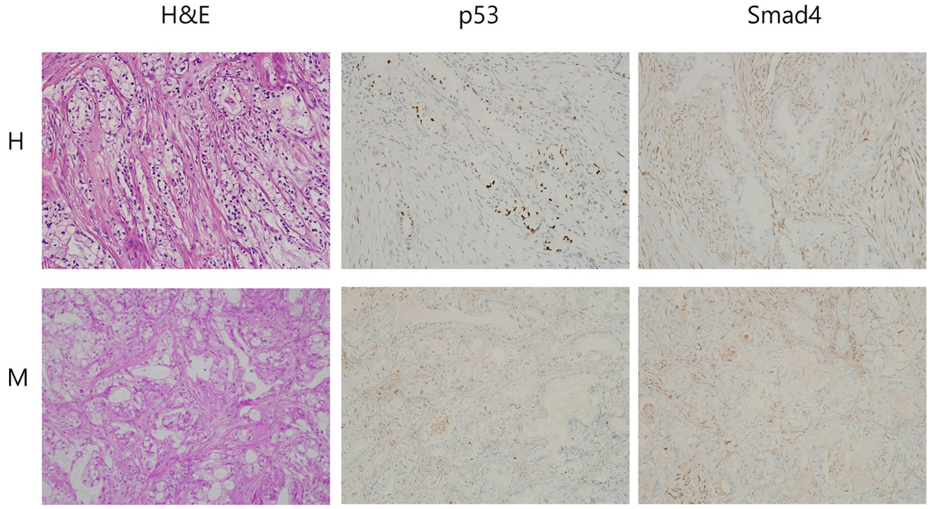

E

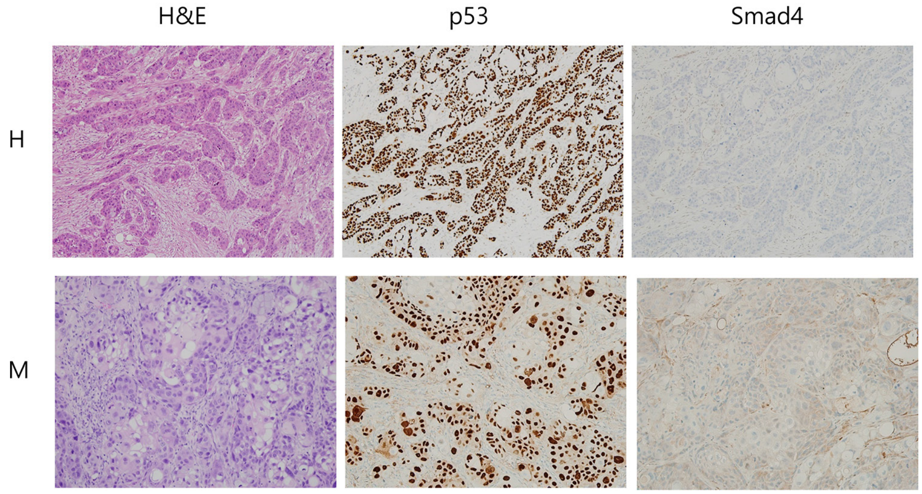

F

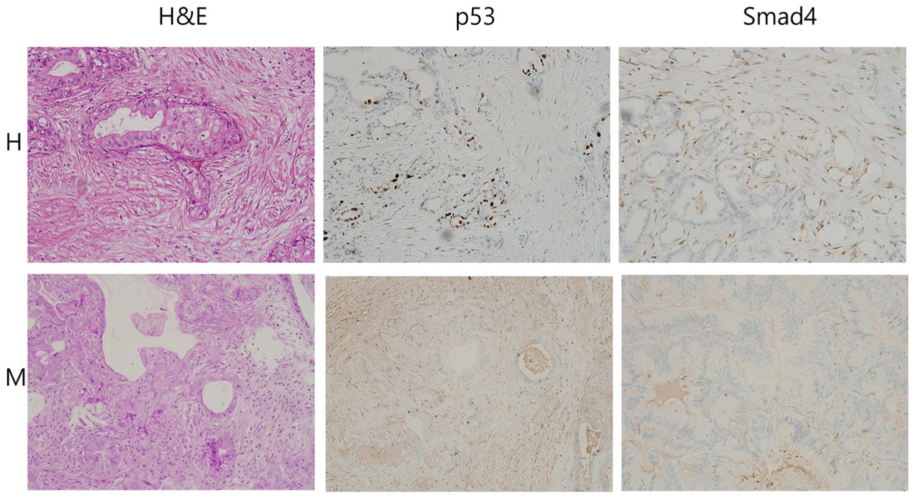

(*Continued*)

G

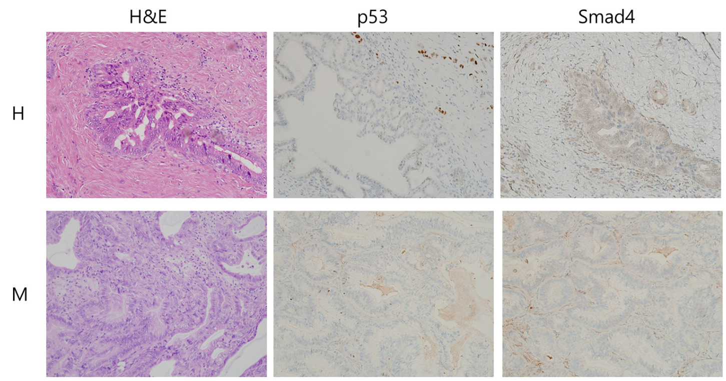

H

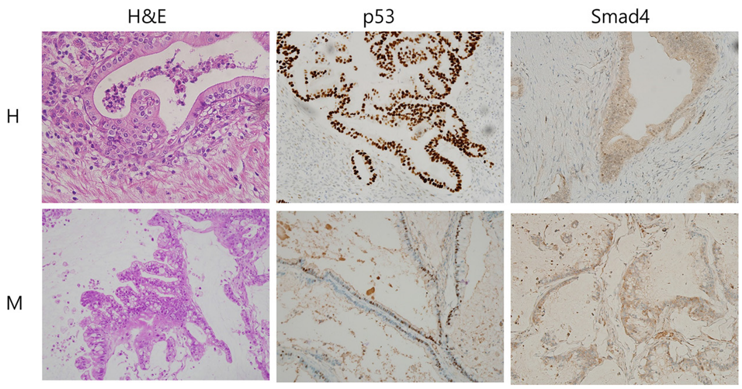

I

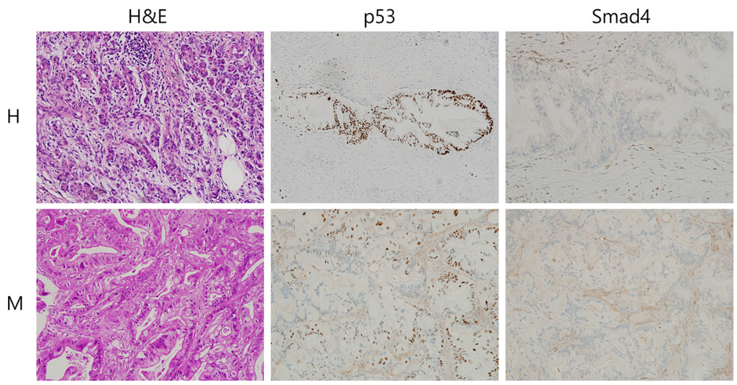

J

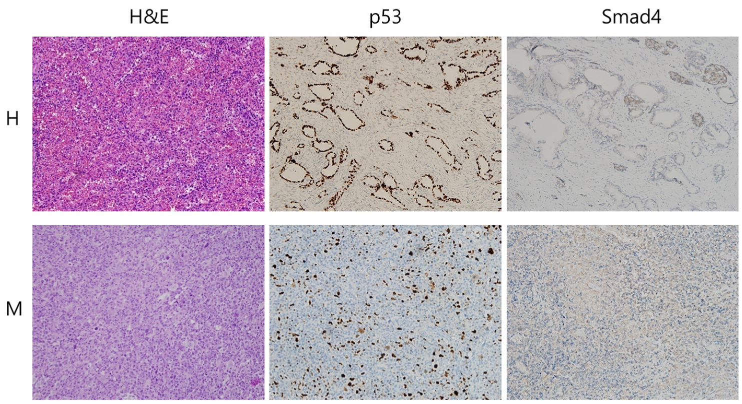

(*Continued*)

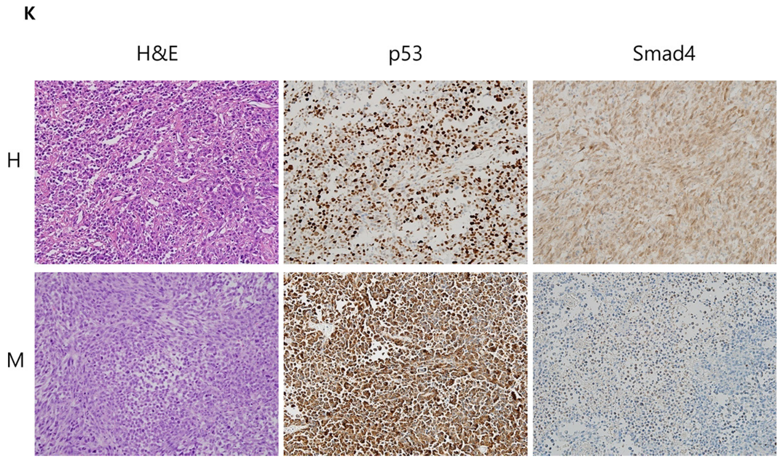

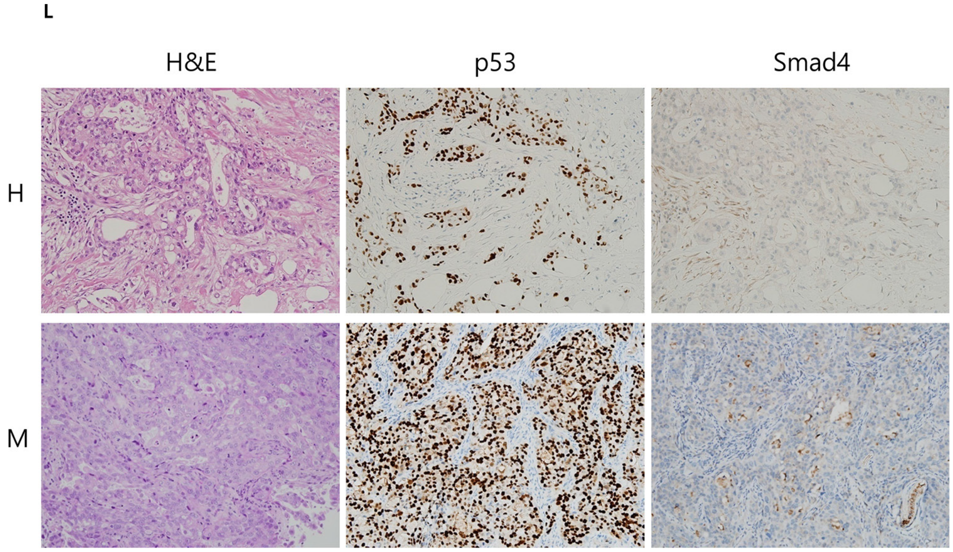

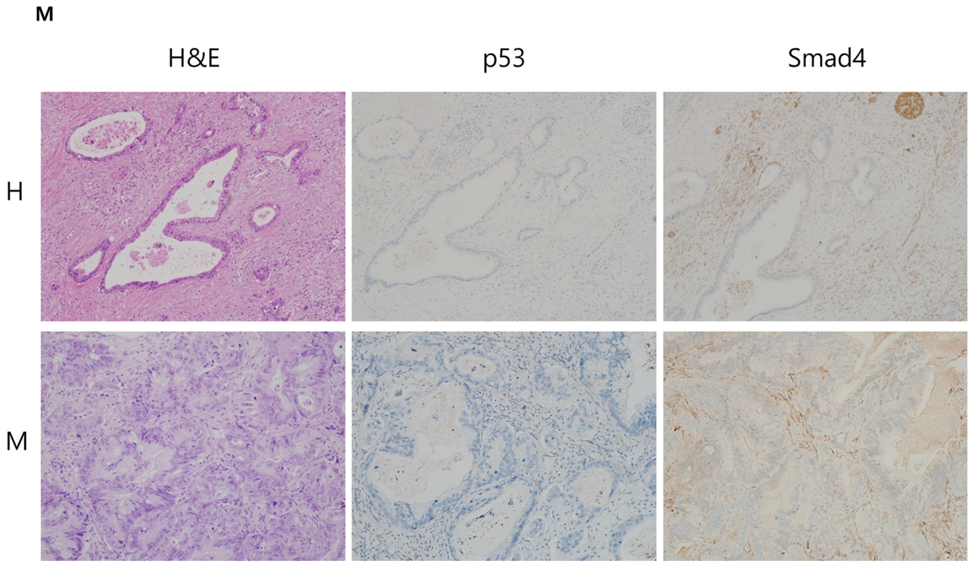

Supplementary Figure S3: A to M. Immunohistochemistry (IHC) or hematoxylin and eosin (H&E) histological data from the primary tumors AMC004 to AMC017 (upper panels; marked as H(Human)) or a patient-derived xenograft (PDX) tumor (lower panels; marked as M(Mouse)). Representative pictures of H&E staining, and p53 and Smad4 IHC are shown.

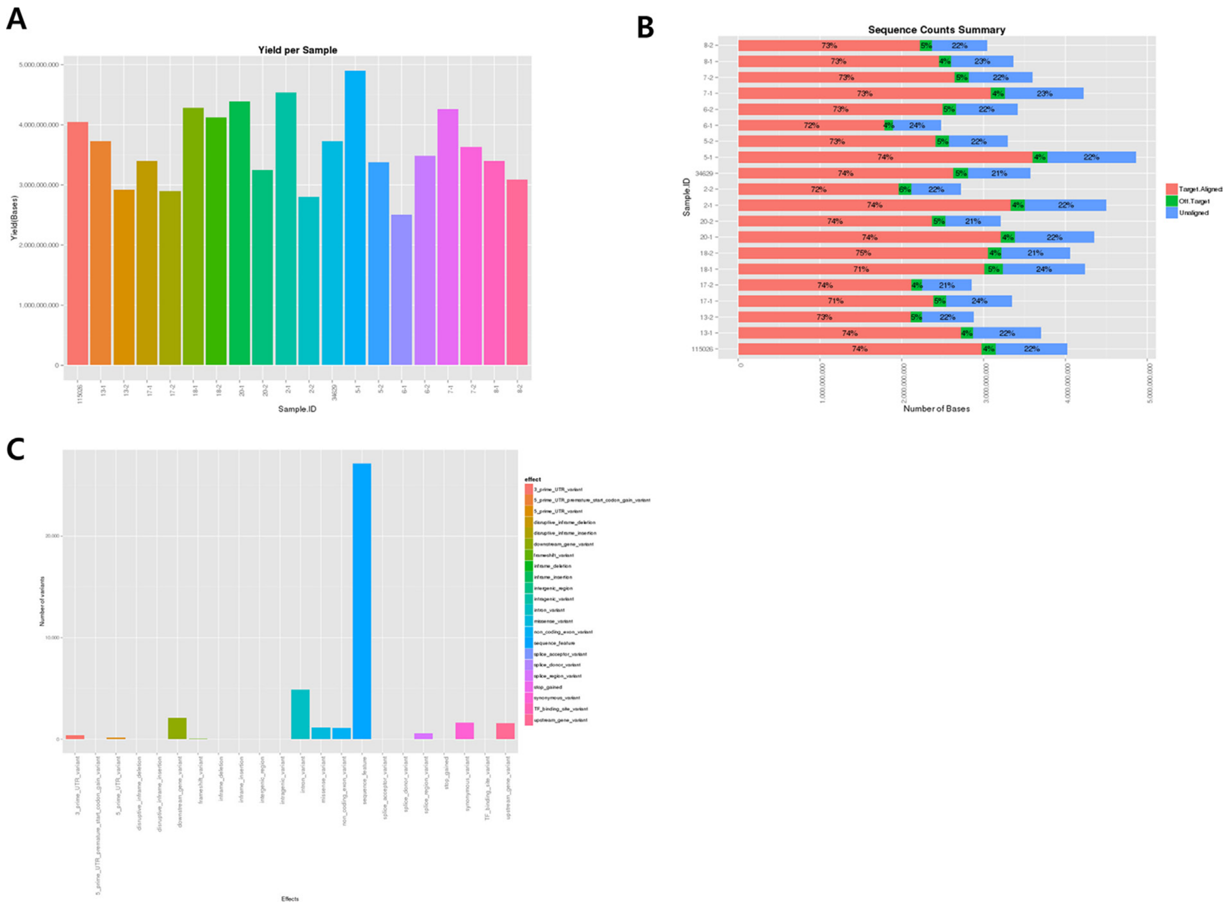

Supplementary Figure S4: Genetic analysis of patient-derived xenograft (PDX) primary tumors using Comprehensive Cancer Panel. A. Graph showing the yield per sample in the Next-Generation Sequencing (NGS) reads. **B.** Sequence Counts Summary showing aligned, off target, and unaligned sequence counts for each of the tumor samples. **C.** Regional annotation of the variants identified in the panel. Note the high number of the sequence_feature (marked as blue) variant type, which is in the protein coding region, due to the data being obtained from the exome sequencing of 402 cancer-related genes.

Supplementary Table S1: Clinical parameters of pancreatic cancer primary cells

Primary cell ID	Gender	Age	Pathological parameters				
			Stage	pT	pN	M	Histology_type
17884	F	48	IIB	3	1	0	Pancreatic ductal adenocarcinoma
19224	M	61	IIB	3	1	0	Pancreatic ductal adenocarcinoma
34629	F	64	IIA	3	0	0	Pancreatic ductal adenocarcinoma
36473	F	59	IIB	3	1	0	Pancreatic sarcomatoid adenocarcinoma*

Five-digit numbers were assigned for identification purposes and the clinical features of each primary cell type are listed.

Supplementary Table S2: List of the genes with single nucleotide polymorphisms (SNPs) that were predicted as being functionally important

See Supplementary File 1

Supplementary Table S3: Functional annotation of the variants

Effect	dbSNP	dbSNP(%)	Novel	Novel(%)	Total
***TOTAL	30,796	10000	10,031	10000	40,827
3_prime_UTR_variant	284	92	100	100	384
5_prime_UTR_premature_start_codon_gain_variant	14	5	0	0	14
5_prime_UTR_variant	97	31	33	33	130
disruptive_inframe_deletion	7	2	16	16	23
disruptive_inframe_insertion	4	1	10	10	14
downstream_gene_variant	1,621	526	490	488	2,111
frameshift_variant	13	4	15	15	28
inframe_deletion	0	0	7	7	7
inframe_insertion	0	0	16	16	16
intergenic_region	1	0	0	0	1
intragenic_variant	0	0	2	2	2
intron_variant	3,441	1120	1,418	1410	4,859
missense_variant	804	261	342	341	1,146
non_coding_exon_variant	867	282	228	227	1,095
sequence_feature	20,612	6690	6,543	6520	27,155
splice_acceptor_variant	22	7	2	2	24
splice_donor_variant	0	0	3	3	3
splice_region_variant	340	110	243	242	583
stop_gained	0	0	15	15	15
synonymous_variant	1,482	481	144	144	1,626
TF_binding_site_variant	6	2	1	1	7
upstream_gene_variant	1,181	383	403	402	1,584
#of variants in coding and UTR					**1804**

UTR: Untranslated region; TF: transcription factor.

Supplementary Table S4: List of the variants that were predicted to be deleterious, as identified using Comprehensive Cancer Panel (CCP) data. REF: reference sequence; ALT: altered sequence; HGVS: Human Genome Variation Society.

See Supplementary File 1

Supplementary Table S5: The number of known variants identified in each of the tumors by Comprehensive Cancer Panel (CCP) analysis

Sample	nSNPs	nInsertions	nDeletions	nComplex	nTotal	nHets	nHomVar	nSingletons	heterozygosity	hetHomRatio
115026	616	30	50	38	734	456	278	23	1.00E-07	1.64
13-1	586	34	51	33	704	398	306	0	1.00E-07	1.3
13-2	584	33	52	32	701	397	304	1	1.00E-07	1.31
17-1	606	39	57	31	733	434	299	6	1.00E-07	1.45
17-2	581	36	52	27	696	330	366	0	1.00E-07	0.9
18-1	554	31	51	37	673	356	317	2	1.00E-07	1.12
18-2	552	33	46	37	668	347	321	0	1.00E-07	1.08
2-1	587	33	50	33	703	398	305	3	1.00E-07	1.3
2-2	561	34	48	33	676	330	346	0	1.00E-07	0.95
20-1	563	33	55	34	685	400	285	16	1.00E-07	1.4
20-2	538	35	50	36	659	308	351	16	1.00E-07	0.88
34629	591	29	52	34	706	389	317	18	1.00E-07	1.23
5-1	581	34	51	35	701	416	285	0	1.00E-07	1.46
5-2	582	34	51	33	700	418	282	1	1.00E-07	1.48
6-1	592	36	54	33	715	411	304	2	1.00E-07	1.35
6-2	541	34	49	36	660	331	329	0	1.00E-07	1.01
7-1	596	34	51	38	719	393	326	0	1.00E-07	1.21
7-2	598	34	46	37	715	385	330	1	1.00E-07	1.17
8-1	635	40	52	35	762	483	279	8	2.00E-07	1.73
8-2	563	40	45	33	681	326	355	0	1.00E-07	0.92

Supplementary Table S6: The number of novel variants identified in each of the tumors by Comprehensive Cancer Panel (CCP) analysis

Sample	nSNPs	nInsertions	nDeletions	nComplex	nTotal	nHets	nHomVar	nSingletons	heterozygosity	hetHomRatio
115026	42	1	15	2	60	57	3	13	0	19
13-1	40	3	15	3	61	57	4	2	0	14.2
13-2	35	3	13	3	54	53	1	3	0	53
17-1	34	2	15	2	53	49	4	0	0	12.2
17-2	33	3	15	4	55	52	3	0	0	17.3
18-1	42	2	18	3	65	63	2	3	0	31.5
18-2	36	2	14	2	54	53	1	0	0	53
2-1	42	4	13	4	63	58	5	4	0	11.6
2-2	29	3	13	3	48	42	6	0	0	7
20-1	36	2	14	2	54	48	6	11	0	8
20-2	31	2	13	3	49	43	6	5	0	7.17
34629	28	1	13	4	46	45	1	8	0	45
5-1	34	4	14	3	55	51	4	0	0	12.8
5-2	35	4	17	3	59	58	1	1	0	58
6-1	32	3	11	3	49	44	5	3	0	8.8
6-2	33	3	16	3	55	49	6	4	0	8.17
7-1	37	2	13	4	56	54	2	3	0	27
7-2	24	2	14	4	44	42	2	0	0	21
8-1	44	1	17	4	66	58	8	1	0	7.25
8-2	42	1	16	2	61	58	3	4	0	19.3

Supplementary Table S7: Coverage of the Comprehensive Cancer Panel (CCP) analysis

Sample	×1(%)	×5(%)	×10(%)	×15(%)	×20(%)	×25(%)	×30(%)	×50(%)	total_bases_ aligned_bases	mean_ coverage_ depth
115026	99.8	99.6	99.5	99.3	99.3	99.2	99.1	98.9	2,978,186,056	1760
13-1	99.8	99.6	99.5	99.4	99.3	99.2	99.2	98.9	2,723,116,470	1610
13-2	99.9	99.7	99.5	99.4	99.3	99.2	99.1	98.9	2,109,245,791	1250
17-1	99.8	99.6	99.5	99.4	99.3	99.2	99.1	98.9	2,389,805,922	1420
17-2	99.8	99.6	99.5	99.3	99.3	99.2	99.1	98.8	2,121,760,748	1260
18-1	99.9	99.7	99.6	99.5	99.4	99.3	99.2	99	3,010,370,819	1780
18-2	99.9	99.8	99.6	99.6	99.5	99.4	99.3	99.1	3,051,246,526	1810
2-1	99.8	99.6	99.5	99.4	99.4	99.3	99.3	99.1	3,330,983,419	1970
2-2	99.8	99.6	99.3	99.1	98.9	98.7	98.6	98.1	1,964,565,898	1160
20-1	99.8	99.6	99.5	99.4	99.3	99.1	99	98.7	3,212,141,634	1900
20-2	99.7	99.5	99.3	99.3	99.2	99.1	99	98.8	2,369,919,932	1400
34629	99.9	99.7	99.6	99.5	99.4	99.3	99.2	99	2,632,180,839	1560
5-1	99.8	99.6	99.5	99.4	99.4	99.3	99.2	99	3,594,801,890	2130
5-2	99.9	99.7	99.6	99.5	99.5	99.4	99.3	99.1	2,416,077,435	1430
6-1	99.8	99.5	99.4	99.2	99	98.9	98.8	98.5	1,787,704,037	1060
6-2	99.9	99.6	99.5	99.4	99.3	99.2	99.2	98.9	2,494,578,771	1480
7-1	99.8	99.6	99.5	99.3	99.3	99.1	99.1	98.8	3,088,396,179	1830
7-2	99.9	99.7	99.6	99.5	99.4	99.3	99.2	99	2,645,211,631	1570
8-1	99.8	99.6	99.4	99.4	99.3	99.2	99.1	98.8	2,457,867,852	1460
8-2	99.8	99.5	99.3	99.1	99	98.9	98.9	98.6	2,224,617,317	1320

Each column indicates the depths, ranging from ×1 to ×50, and each cell represents the proportion of genes with a higher depth than indicated.
