# Supplementary material for: Generation and molecular characterization of pancreatic cancer patient-derived xenografts reveals their heterologous nature
Source: Oncotarget. 2016 Aug 23;7(38):62533–46. doi: 10.18632/oncotarget.11530 (PMC5308744; doi:10.18632/oncotarget.11530)
Supplement: Supplementary file 2 [file oncotarget-07-62533-s002.docx]

**Supplementary Table S2. List of the genes with single nucleotide polymorphisms (SNPs) that were predicted as being functionally important**

| **Gene name** | | | | | | | | | | |
| --- | --- | --- | --- | --- | --- | --- | --- | --- | --- | --- |
| *DNAAF2* | *C8orf31* | *C1orf65* | *DOPEY1* | *GTPBP3* | *CCRL2* | *P4HA3* | *SARDH* | *SBF2* | *CPN2* | *SVEP1* |
| *TESPA1* | *TCF7L2* | *SCRIB* | *GBP3* | *ONECUT2* | *RGS22* | *KRT76* | *NGFR* | *OR4X1* | *CHMP1A* | *NXPE1* |
| *PKD1L2* | *MYOM2* | *FAM71F2* | *TTC9* | *MOCOS* | *TCF7* | *C15orf26* | *TMEM176B* | *EGFLAM* | *HIPK4* | *FGFBP3* |
| *MYCBPAP* | *OR7D2* | *GOLGA1* | *FAM129C* | *ACOX1* | *FAM194B* | *DUSP6* | *PRSS21* | *KLHL38* | *TBX6* | *C10orf71* |
| *KIAA0753* | *OR4C46* | *NFATC3* | *CDC20B* | *PAK6* | *MYO15A* | *CCDC8* | *OR2F1* | *KCNU1* | *C14orf159* | *MPDU1* |
| *OR5H6* | *MYO10* | *PRPH* | *OR52L1* | *MIR548H4* | *FAM198A* | *EVC* | *PMEL* | *ANKRD6* | *RIC8A* | *OR51B2* |
| *BTBD16* | *OR10G9* | *TRAPPC11* | *MYOM3* | *NOX5* | *ALDH1L1* | *PITRM1* | *TIAM1* | *ADAMTS16* | *CASKIN2* | *OR51G1* |
| *RGS11* | *LOC100505666* | *TAS2R7* | *PRIM2* | *SPESP1* | *SERPINA9* | *QRICH2* | *HCLS1* | *MROH2B* | *CARD14* | *HELT* |
| *KRTAP11-1* | *ADAM15* | *KRT3* | *ITGA1* | *RIN3* | *KRT83* | *KIAA1524* | *TIPIN* | *TAF1B* | *RAI1* | *HUNK* |
| *TPPP2* | *OR1A1* | *NIN* | *TDG* | *TSKU* | *NAV1* | *OXER1* | *CYP4A22* | *TTN* | *WDR90* | *WNK2* |
| *TRIOBP* | *PUS1* | *OR2K2* | *VN1R5* | *PALD1* | *RABL6* | *DMBT1* | *CYP4F8* | *STK36* | *PLA2G4D* | *ARSB* |
| *LMO7* | *ZNF30* | *C3orf70* | *FAM53B* | *TTF1* | *HEATR1* | *AQP11* | *TNXB* | *07-Mar* | *OR6S1* | *CFH* |
| *CCDC169-SOHLH2* | *TBRG1* | *USP6* | *TEX13B* | *NIPSNAP3B* | *FTSJ3* | *MRPL37* | *AKAP3* | *OR5H2* | *TEKT1* | *MTCH2* |
| *CCDC169* | *ADNP* | *AZI1* | *USP45* | *ZNF484* | *ZNF761* | *NIPSNAP3A* | *TTLL6* | *RGL3* | *LRRN2* | *C7* |
| *KCNK4* | *OR5D14* | *ACTBL2* | *OR1J2* | *MTUS1* | *ACAT2* | *ABCC4* | *FMO2* | *TAS2R42* | *ICOSLG* | *KIF20B* |
| *VARS2* | *SGSH* | *F5* | *AFAP1-AS1* | *RRS1* | *RNF43* | *GALNT2* | *PASK* | *OR4D6* | *SP110* | *CBR3-AS1* |
| *PLCD1* | *IGSF10* | *FAM118A* | *AFAP1* | *LRRFIP1* | *USP35* | *TADA1* | *SIGLEC6* | *ZNF229* | *CCDC137* | *CBR3* |
| *TIAM2* | *KRT36* | *FEZ1* | *BAG3* | *PRUNE* | *MMP27* | *PADI3* | *USP31* | *LOC100506134* | *LTBP3* | *TCF3* |
| *MCPH1* | *MROH5* | *TRIP11* | *CRNN* | *TRIM45* | *OR52J3* | *MGAM* | *MRC1* | *TTC21B* | *GGH* | *CPVL* |
| *COL11A2* | *IL1RL2* | *GPR112* | *PRICKLE1* | *COL9A2* | *PPP2R4* | *ADARB2* | *ACIN1* | *SHBG* | *MYEOV* | *PLA2G7* |
| *TRPA1* | *DOCK5* | *ILVBL* | *PLEKHH1* | *PHF21A* | *OR8D2* | *KDM6B* | *IL27* | *SAT2* | *CAPG* | *BTD* |
| *HKDC1* | *AP1G2* | *COL6A6* | *IQGAP2* | *PPARGC1A* | *PDLIM5* | *CPT1B* | *OR10H2* | *CDC6* | *VARS* | *GPC4* |
| *AP1G1* | *CCDC149* | *F2* | *IFI44L* | *ZMIZ2* | *SLC22A14* | *CHKB-CPT1B* | *RPGR* | *CCNJL* | *SLC2A7* | *FGF2* |
| *SUCLG2* | *NMBR* | *RAB11FIP2* | *KIAA1751* | *TRIM31* | *SLC22A20* | *CHIA* | *ETFA* | *APOB* | *ACO2* | *NUDT6* |
| *OCEL1* | *PLOD3* | *RALGPS1* | *MICA* | *IMPG1* | *CD320* | *MATN2* | *IRS1* | *WBSCR27* | *POLR3H* | *FUT2* |
| *SURF6* | *AMBN* | *BPI* | *MYBPC1* | *BANK1* | *PDLIM7* | *TRAK2* | *CCR5* | *TRIML1* | *CRTAP* | *PLEKHG2* |
| *CHI3L1* | *OR56B1* | *PCDHGA9* | *EML2* | *EFCC1* | *GRID1* | *PYCRL* | *PPP1R3A* | *TET1* | *OR2G2* | *C6orf10* |
| *NRAP* | *ARSD* | *PCDHGB6* | *DCAF4* | *GTF2E1* | *C4orf26* | *ZNRF4* | *XRCC1* | *INHA* | *MEFV* | *LCTL* |
| *FPR1* | *OR1I1* | *PCDHGA10* | *BOC* | *CX3CR1* | *SVOPL* | *GRIN3B* | *MMP7* | *FAM188B* | *PLEKHG5* | *NEK4* |
| *FEZ2* | *TAPBPL* | *PCDHGB7* | *FAM171A1* | *MPHOSPH10* | *C19orf57* | *COBL* | *FUK* | *NFXL1* | *SLC15A2* | *SAMD9* |
| *NBR1* | *C1orf127* | *PCDHGA11* | *C1R* | *PRR5* | *LTBP2* | *C2CD2L* | *ZKSCAN3* | *MYH15* | *PPAP2C* | *FBXO34* |
| *PGLYRP3* | *FAM149B1* | *ERI2* | *HHIPL2* | *PRR5-ARHGAP8* | *SLC52A1* | *KCTD20* | *CTU1* | *VPS13C* | *AKAP11* | *IDO2* |
| *IGF2R* | *CCP110* | *ACSM3* | *MOV10L1* | *AK1* | *CEP120* | *SNAP47* | *PIK3CG* | *CLDN23* | *KLRB1* | *AQPEP* |
| *ALDH1A1* | *CWC22* | *C15orf55* | *ALPK2* | *TBC1D1* | *TTLL5* | *RBBP8NL* | *KRT39* | *DFNA5* | *DLG1* | *SLC7A9* |
| *SLC28A1* | *ZNF195* | *MYBBP1A* | *HSF5* | *AOC2* | *LNPEP* | *NKAIN4* | *DSC1* | *PARP15* | *DDX51* |  |
| *NELL1* | *C9orf171* | *C3orf30* | *TNS1* | *ENPEP* | *SPINK5* | *IRF6* | *MAP4K5* | *IL17RA* | *C19orf45* |  |
| *ABO* | *FAM71F1* | *BIVM-ERCC5* | *02-Mar* | *NKTR* | *EHHADH* | *PNMA2* | *MAPT-AS1* | *TMC6* | *CAMKK2* |  |
| *DNAJB11* | *RSPH4A* | *ERCC5* | *ACTRT2* | *PDZRN4* | *C9orf50* | *HEMK1* | *SPPL2C* | *PGAM5* | *CPOX* |  |
| *IGSF5* | *LOC100652739* | *KHDC3L* | *USP29* | *CDH11* | *RPGRIP1L* | *PRMT6* | *C2orf71* | *LRRC55* | *NQO1* |  |
| *ZNF519* | *RAET1E* | *NME8* | *GORASP2* | *CHURC1-FNTB* | *BST1* | *CCT3* | *WDR72* | *TMPRSS2* | *HDHD1* |  |
| *C14orf80* | *RNU6-28* | *MAST3* | *ALDH5A1* | *RAB15* | *EZH2* | *PEPD* | *SH3TC2* | *CDON* | *DDX27* |  |
| *OR6K2* | *TP53BP1* | *EIF1B-AS1* | *DHDH* | *MUSK* | *MINK1* | *CTSS* | *NOTCH4* | *HEXA* | *PDCD6IP* |  |
| *OR6C65* | *PCDHGA1* | *MYRIP* | *INADL* | *KIAA0141* | *SLC14A1* | *ZNF140* | *PLCE1* | *CCDC116* | *GFAP* |  |
| *C11orf82* | *PCDHGA2* | *CHRNA9* | *KIAA1210* | *NUAK2* | *YDJC* | *GLI1* | *H6PD* | *LRRC71* | *IQGAP3* |  |
| *OR5AN1* | *PCDHGA3* | *GPC5* | *PRODH2* | *LRP10* | *C9orf131* | *PYGB* | *FSIP2* | *OR10J1* | *GDPD4* |  |
| *NUGGC* | *PCDHGB1* | *LMF1* | *SCARF1* | *COL16A1* | *ITGA8* | *NT5DC1* | *ZNF276* | *SPATA3* | *GRID2IP* |  |
| *OR5D13* | *PCDHGA4* | *TPSG1* | *SPG11* | *NEK5* | *COL15A1* | *COL10A1* | *FANCA* | *CGN* | *OR10Q1* |  |
| *MPEG1* | *PCDHGB2* | *MTA1* | *CD101* | *SPEF2* | *ANKRD16* | *HIVEP1* | *SPAG17* | *PON2* | *KIAA1377* |  |
| *NHLRC2* | *PCDHGA5* | *TPO* | *EMR2* | *C1QTNF3-AMACR* | *SWT1* | *PDCD1* | *VWDE* | *ANKRD30A* | *LRIT1* |  |
| *MTMR6* | *PCDHGB3* | *OR2B11* | *LOC100131691* | *AMACR* | *CHMP4A* | *SULT1C3* | *CERS4* | *KDELR3* | *SLCO1C1* |  |
| *OR8U8* | *PCDHGA6* | *AHSG* | *MZF1* | *GUCY2D* | *HHAT* | *PCDH15* | *DLGAP5* | *C7orf31* | *COL24A1* |  |
| *OR5AR1* | *PCDHGA7* | *SYT8* | *MTHFSD* | *TNFRSF13B* | *TOP1MT* | *PNLIPRP3* | *ATP5S* | *PPARGC1B* | *RNASET2* |  |
| *MRM1* | *PCDHGB4* | *MAGEB16* | *CACNA1H* | *COL4A3* | *RP1* | *ZNF546* | *KIF4B* | *GPR101* | *TLR10* |  |
| *FCN2* | *PCDHGA8* | *ACTL9* | *SCUBE2* | *UROC1* | *IQCE* | *CYP4F2* | *DKK2* | *CASC5* | *MAGEC3* |  |
| *TMCC1* | *PCDHGB5* | *AGT* | *DSPP* | *SPNS3* | *KRT71* | *APOL5* | *CAPN13* | *TMPRSS4* | *B4GALNT3* |  |
| *ARAP2* | *C3orf20* | *CDH23* | *TNK2* | *SHROOM3* | *PDIA4* | *LAMB3* | *EGFL6* | *SH3RF3* | *OLR1* |  |
| *GGA3* | *IL17RD* | *KLF10* | *PPAN-P2RY11* | *TNRC6C* | *DKKL1* | *E2F2* | *MYZAP* | *TECPR1* | *OR51B5* |  |
| *KRT13* | *PLB1* | *INMT-FAM188B* | *P2RY11* | *ZSCAN5A* | *BMP2K* | *NOD1* | *GCOM1* | *CELSR2* | *OR51I2* |  |
| *TTC29* | *ALDH2* | *INMT* | *IL4I1* | *FAM170A* | *MTRR* | *LAMB4* | *VPS53* | *FBRSL1* | *IGSF22* |  |
| *HTR3D* | *TTC24* | *ERV3-1* | *NUP62* | *PZP* | *WDR55* | *ABCB5* | *RECK* | *TMC8* | *MKI67* |  |

**Supplementary Table S4. List of the variants that were predicted to be deleterious, as identified using Comprehensive Cancer Panel (CCP) data**

| **CHROM** | **POS** | **ID** | **REF** | **ALT** | **Variant Type** | **Allele** | **Effect** | **Impact** | **Gene_Name** | **Transcript_BioType** | **Rank** | **HGVS.c** | **Num Samples Affected** | **Num Homo Ref** | **Num Hetero** | **Num Homo Alt** |
| --- | --- | --- | --- | --- | --- | --- | --- | --- | --- | --- | --- | --- | --- | --- | --- | --- |
| chr1 | 45797760 | rs77542170 | T | C | SNP | C | splice_acceptor_variant&intron_variant | HIGH | `MUTYH | protein_coding | `10/15 | c.934-2A>G | 2 | 18 | 2 | 0 |
| chr1 | 45797760 | rs77542170 | T | C | SNP | C | splice_acceptor_variant&intron_variant | HIGH | `MUTYH | protein_coding | `11/16 | c.850-2A>G | 2 | 18 | 2 | 0 |
| chr1 | 45797760 | rs77542170 | T | C | SNP | C | splice_acceptor_variant&intron_variant | HIGH | `MUTYH | protein_coding | `10/15 | c.883-2A>G | 2 | 18 | 2 | 0 |
| chr1 | 45797760 | rs77542170 | T | C | SNP | C | splice_acceptor_variant&intron_variant | HIGH | `MUTYH | protein_coding | `10/15 | c.850-2A>G | 2 | 18 | 2 | 0 |
| chr1 | 45797760 | rs77542170 | T | C | SNP | C | splice_acceptor_variant&intron_variant | HIGH | `MUTYH | protein_coding | `10/15 | c.853-2A>G | 2 | 18 | 2 | 0 |
| chr1 | 45797760 | rs77542170 | T | C | SNP | C | splice_acceptor_variant&intron_variant | HIGH | `MUTYH | nonsense_mediated_decay | `10/15 | n.*663-2A>G | 2 | 18 | 2 | 0 |
| chr1 | 45797760 | rs77542170 | T | C | SNP | C | splice_acceptor_variant&intron_variant | HIGH | `MUTYH | protein_coding | `10/15 | c.850-2A>G | 2 | 18 | 2 | 0 |
| chr1 | 45797760 | rs77542170 | T | C | SNP | C | splice_acceptor_variant&intron_variant | HIGH | `MUTYH | protein_coding | `10/15 | c.925-2A>G | 2 | 18 | 2 | 0 |
| chr1 | 45797760 | rs77542170 | T | C | SNP | C | splice_acceptor_variant&intron_variant | HIGH | `MUTYH | protein_coding | `10/15 | c.895-2A>G | 2 | 18 | 2 | 0 |
| chr1 | 45797760 | rs77542170 | T | C | SNP | C | splice_acceptor_variant&intron_variant | HIGH | `MUTYH | nonsense_mediated_decay | `10/15 | n.*663-2A>G | 2 | 18 | 2 | 0 |
| chr1 | 45797760 | rs77542170 | T | C | SNP | C | splice_acceptor_variant&intron_variant | HIGH | `MUTYH | protein_coding | `10/15 | c.892-2A>G | 2 | 18 | 2 | 0 |
| chr1 | 45797760 | rs77542170 | T | C | SNP | C | splice_acceptor_variant&intron_variant | HIGH | `MUTYH | protein_coding | `10/15 | c.883-2A>G | 2 | 18 | 2 | 0 |
| chr1 | 45797760 | rs77542170 | T | C | SNP | C | splice_acceptor_variant&intron_variant | HIGH | `MUTYH | nonsense_mediated_decay | `2/7 | n.244-2A>G | 2 | 18 | 2 | 0 |
| chr1 | 45797760 | rs77542170 | T | C | SNP | C | splice_acceptor_variant&intron_variant | HIGH | `MUTYH | protein_coding | `1/5 | c.112-2A>G | 2 | 18 | 2 | 0 |
| chr1 | 45797760 | rs77542170 | T | C | SNP | C | splice_acceptor_variant&intron_variant | HIGH | `MUTYH | nonsense_mediated_decay | `4/9 | n.*179-2A>G | 2 | 18 | 2 | 0 |
| chr1 | 45797760 | rs77542170 | T | C | SNP | C | splice_acceptor_variant&intron_variant | HIGH | `MUTYH | protein_coding | `10/15 | c.892-2A>G | 2 | 18 | 2 | 0 |
| chr1 | 45797760 | rs77542170 | T | C | SNP | C | splice_acceptor_variant&intron_variant | HIGH | `MUTYH | protein_coding | `5/6 | c.466-2A>G | 2 | 18 | 2 | 0 |
| chr1 | 45797760 | rs77542170 | T | C | SNP | C | splice_acceptor_variant&intron_variant | HIGH | `MUTYH | retained_intron | `4/4 | n.541-2A>G | 2 | 18 | 2 | 0 |
| chr1 | 45797760 | rs77542170 | T | C | SNP | C | splice_acceptor_variant&intron_variant | HIGH | `MUTYH | retained_intron | `1/2 | n.215-2A>G | 2 | 18 | 2 | 0 |
| chr1 | 45797760 | rs77542170 | T | C | SNP | C | splice_acceptor_variant&intron_variant | HIGH | `MUTYH | nonsense_mediated_decay | `8/8 | n.*179-2A>G | 2 | 18 | 2 | 0 |
| chr1 | 45797760 | rs77542170 | T | C | SNP | C | splice_acceptor_variant&intron_variant | HIGH | `MUTYH | nonsense_mediated_decay | `9/9 | n.*589-2A>G | 2 | 18 | 2 | 0 |
| chr1 | 1.45E+08 | rs369106087 | GT | G | DEL | G | frameshift_variant | HIGH | `PDE4DIP | protein_coding | `9/47 | c.1140delA | 18 | 2 | 18 | 0 |
| chr1 | 1.45E+08 | rs369106087 | GT | G | DEL | G | frameshift_variant | HIGH | `PDE4DIP | protein_coding | `9/46 | c.927delA | 18 | 2 | 18 | 0 |
| chr1 | 1.45E+08 | rs369106087 | GT | G | DEL | G | frameshift_variant | HIGH | `PDE4DIP | protein_coding | `6/44 | c.729delA | 18 | 2 | 18 | 0 |
| chr1 | 1.45E+08 | rs369106087 | GT | G | DEL | G | frameshift_variant | HIGH | `PDE4DIP | protein_coding | `6/44 | c.729delA | 18 | 2 | 18 | 0 |
| chr1 | 1.45E+08 | rs369106087 | GT | G | DEL | G | frameshift_variant | HIGH | `PDE4DIP | protein_coding | `9/46 | c.1140delA | 18 | 2 | 18 | 0 |
| chr1 | 1.45E+08 | rs369106087 | GT | G | DEL | G | frameshift_variant | HIGH | `PDE4DIP | protein_coding | `6/22 | c.729delA | 18 | 2 | 18 | 0 |
| chr1 | 1.45E+08 | rs369106087 | GT | G | DEL | G | frameshift_variant | HIGH | `PDE4DIP | protein_coding | `6/23 | c.729delA | 18 | 2 | 18 | 0 |
| chr1 | 1.45E+08 | rs369106087 | GT | G | DEL | G | frameshift_variant | HIGH | `PDE4DIP | protein_coding | `2/19 | c.1218delA | 18 | 2 | 18 | 0 |
| chr1 | 1.45E+08 | rs369106087 | GT | G | DEL | G | frameshift_variant | HIGH | `PDE4DIP | protein_coding | `2/17 | c.1218delA | 18 | 2 | 18 | 0 |
| chr1 | 1.45E+08 | rs369106087 | GT | G | DEL | G | frameshift_variant | HIGH | `PDE4DIP | protein_coding | `2/17 | c.90delA | 18 | 2 | 18 | 0 |
| chr2 | 85536533 | . | TC | T | DEL |  | frameshift_variant | HIGH | `TCF7L1 | protein_coding | `12/12 | c.1718delC | 2 | 18 | 2 | 0 |
| chr3 | 37818889 | . | C | T | SNP | T | stop_gained | HIGH | `ITGA9 | protein_coding | `24/28 | c.2548C>T | 2 | 18 | 2 | 0 |
| chr5 | 38496637 | rs3729740 | C | T | SNP | T | splice_acceptor_variant&intron_variant | HIGH | `LIFR | nonsense_mediated_decay | `2/6 | n.109-1G>A | 4 | 16 | 2 | 2 |
| chr5 | 55243415 | . | G | A | SNP | A | stop_gained&splice_region_variant | HIGH | `IL6ST | protein_coding | `13/15 | c.1843C>T | 13 | 7 | 13 | 0 |
| chr5 | 55243415 | . | G | A | SNP | A | stop_gained&splice_region_variant | HIGH | `IL6ST | protein_coding | `15/17 | c.1843C>T | 13 | 7 | 13 | 0 |
| chr5 | 55243415 | . | G | A | SNP | A | stop_gained&splice_region_variant | HIGH | `IL6ST | protein_coding | `14/16 | c.1843C>T | 13 | 7 | 13 | 0 |
| chr5 | 55243415 | . | G | A | SNP | A | stop_gained&splice_region_variant | HIGH | `IL6ST | protein_coding | `12/14 | c.1660C>T | 13 | 7 | 13 | 0 |
| chr6 | 93956653 | . | G | T | SNP | T | stop_gained | HIGH | `EPHA7 | protein_coding | `15/17 | c.2583C>A | 14 | 5 | 12 | 2 |
| chr8 | 48805816 | rs11411516 | A | AG | INS | AG | frameshift_variant&splice_region_variant | HIGH | `PRKDC | protein_coding | `31/87 | c.3729dupC | 20 | 0 | 0 | 20 |
| chr8 | 48805816 | rs11411516 | A | AG | INS | AG | frameshift_variant&splice_region_variant | HIGH | `PRKDC | protein_coding | `31/86 | c.3729dupC | 20 | 0 | 0 | 20 |
| chr8 | 1.46E+08 | . | A | C | SNP | C | splice_donor_variant&intron_variant | HIGH | `RECQL4 | protein_coding | `15/21 | c.2463+2T>G | 7 | 13 | 7 | 0 |
| chr8 | 1.46E+08 | . | A | C | SNP | C | splice_donor_variant&intron_variant | HIGH | `RECQL4 | processed_transcript | `13/19 | n.2590+2T>G | 7 | 13 | 7 | 0 |
| chr8 | 1.46E+08 | . | A | C | SNP | C | splice_donor_variant&intron_variant | HIGH | `RECQL4 | processed_transcript | `14/20 | n.2506+2T>G | 7 | 13 | 7 | 0 |
| chr8 | 1.46E+08 | rs11342077 | CG | C | DEL | C | frameshift_variant&splice_region_variant | HIGH | `RECQL4 | protein_coding | `15/22 | c.2296delC | 20 | 0 | 0 | 20 |
| chr9 | 1.34E+08 | . | A | AC | INS | AC | frameshift_variant | HIGH | `NUP214 | protein_coding | `29/36 | c.4484_4485insC | 18 | 2 | 8 | 10 |
| chr9 | 1.34E+08 | . | A | AC | INS | AC | frameshift_variant | HIGH | `NUP214 | protein_coding | `29/36 | c.4481_4482insC | 18 | 2 | 8 | 10 |
| chr9 | 1.34E+08 | . | A | AC | INS | AC | frameshift_variant | HIGH | `NUP214 | protein_coding | `29/36 | c.4451_4452insC | 18 | 2 | 8 | 10 |
| chr9 | 1.34E+08 | . | A | AC | INS | AC | frameshift_variant | HIGH | `NUP214 | protein_coding | `4/11 | c.959_960insC | 18 | 2 | 8 | 10 |
| chr9 | 1.34E+08 | . | A | AC | INS | AC | frameshift_variant | HIGH | `NUP214 | protein_coding | `2/2 | c.812_813insC | 18 | 2 | 8 | 10 |
| chr10 | 89720633 | . | CT | C,CTT | DEL,INS | T | splice_acceptor_variant&intron_variant | HIGH | `PTEN | protein_coding | `7/8 | c.802-3dupT | 20 | 0 | 20 | 0 |
| chr10 | 89720633 | . | CT | C,CTT | DEL,INS | T | splice_acceptor_variant&intron_variant | HIGH | `PTEN | processed_transcript | `2/2 | n.229-3dupT | 20 | 0 | 20 | 0 |
| chr17 | 7579470 | . | C | CG | INS | CG | frameshift_variant | HIGH | `TP53 | protein_coding | `4/11 | c.216dupC | 8 | 12 | 7 | 1 |
| chr17 | 7579470 | . | C | CG | INS | CG | frameshift_variant | HIGH | `TP53 | protein_coding | `3/7 | c.216dupC | 8 | 12 | 7 | 1 |
| chr17 | 7579470 | . | C | CG | INS | CG | frameshift_variant | HIGH | `TP53 | protein_coding | `3/9 | c.216dupC | 8 | 12 | 7 | 1 |
| chr17 | 7579470 | . | C | CG | INS | CG | frameshift_variant | HIGH | `TP53 | protein_coding | `4/12 | c.216dupC | 8 | 12 | 7 | 1 |
| chr17 | 7579470 | . | C | CG | INS | CG | frameshift_variant | HIGH | `TP53 | protein_coding | `4/12 | c.216dupC | 8 | 12 | 7 | 1 |
| chr17 | 7579470 | . | C | CG | INS | CG | frameshift_variant | HIGH | `TP53 | protein_coding | `4/11 | c.216dupC | 8 | 12 | 7 | 1 |
| chr17 | 7579470 | . | C | CG | INS | CG | frameshift_variant | HIGH | `TP53 | protein_coding | `4/5 | c.216dupC | 8 | 12 | 7 | 1 |
| chr17 | 7579470 | . | C | CG | INS | CG | frameshift_variant | HIGH | `TP53 | protein_coding | `4/5 | c.216dupC | 8 | 12 | 7 | 1 |
| chr17 | 7579470 | . | C | CG | INS | CG | frameshift_variant | HIGH | `TP53 | protein_coding | `5/6 | c.216dupC | 8 | 12 | 7 | 1 |
| chr18 | 48573537 | . | G | T | SNP | T | stop_gained | HIGH | `SMAD4 | protein_coding | `2/12 | c.121G>T | 1 | 19 | 1 | 0 |
| chr18 | 48573537 | . | G | T | SNP | T | stop_gained | HIGH | `SMAD4 | protein_coding | `3/9 | c.121G>T | 1 | 19 | 1 | 0 |
| chr18 | 48573537 | . | G | T | SNP | T | stop_gained | HIGH | `SMAD4 | protein_coding | `2/3 | c.121G>T | 1 | 19 | 1 | 0 |
| chr18 | 48573537 | . | G | T | SNP | T | stop_gained | HIGH | `SMAD4 | protein_coding | `2/12 | c.121G>T | 1 | 19 | 1 | 0 |
| chr18 | 48573537 | . | G | T | SNP | T | stop_gained | HIGH | `SMAD4 | protein_coding | `2/3 | c.121G>T | 1 | 19 | 1 | 0 |
| chr18 | 48573537 | . | G | T | SNP | T | stop_gained | HIGH | `SMAD4 | protein_coding | `2/3 | c.121G>T | 1 | 19 | 1 | 0 |
| chr18 | 48573537 | . | G | T | SNP | T | stop_gained | HIGH | `SMAD4 | protein_coding | `2/4 | c.121G>T | 1 | 19 | 1 | 0 |
| chr18 | 48573537 | . | G | T | SNP | T | stop_gained | HIGH | `SMAD4 | protein_coding | `2/3 | c.121G>T | 1 | 19 | 1 | 0 |
